# NDRG1 acts as an oncogene in triple-negative breast cancer and its loss sensitizes cells to mitochondrial iron chelation

**DOI:** 10.3389/fphar.2024.1422369

**Published:** 2024-06-25

**Authors:** Sukanya B. Jadhav, Michaela Vondrackova, Petra Potomova, Cristian Sandoval-Acuña, Jana Smigova, Kristyna Klanicova, Daniel Rosel, Jan Brabek, Jan Stursa, Lukas Werner, Jaroslav Truksa

**Affiliations:** ^1^ Institute of Biotechnology of the Czech Academy of Sciences, BIOCEV Research Centre, Vestec, Czechia; ^2^ Faculty of Sciences, Charles University, Prague, Czechia; ^3^ Faculty of Sciences, BIOCEV Research Centre, Charles University, Vestec, Czechia

**Keywords:** breast cancer, NDRG1, mitochondrial iron chelation, oncogene, tumor suppressor, GSK3α/β, mitoDFO, mitoDFX

## Abstract

Multiple studies indicate that iron chelators enhance their anti-cancer properties by inducing NDRG1, a known tumor and metastasis suppressor. However, the exact role of NDRG1 remains controversial, as newer studies have shown that NDRG1 can also act as an oncogene. Our group recently introduced mitochondrially targeted iron chelators deferoxamine (mitoDFO) and deferasirox (mitoDFX) as effective anti-cancer agents. In this study, we evaluated the ability of these modified chelators to induce NDRG1 and the role of NDRG1 in breast cancer. We demonstrated that both compounds specifically increase NDRG1 without inducing other NDRG family members. We have documented that the effect of mitochondrially targeted chelators is at least partially mediated by GSK3α/β, leading to phosphorylation of NDRG1 at Thr^346^ and to a lesser extent on Ser^330^. Loss of *NDRG1* increases cell death induced by mitoDFX. Notably, MDA-MB-231 cells lacking NDRG1 exhibit reduced extracellular acidification rate and grow slower than parental cells, while the opposite is true for ER+ MCF7 cells. Moreover, overexpression of full-length NDRG1 and the N-terminally truncated isoform (59112) significantly reduced sensitivity towards mitoDFX in ER+ cells. Furthermore, cells overexpressing full-length NDRG1 exhibited a significantly accelerated tumor formation, while its N-terminally truncated isoforms showed significantly impaired capacity to form tumors. Thus, overexpression of full-length NDRG1 promotes tumor growth in highly aggressive triple-negative breast cancer.

## 1 Introduction

Metastasis and invasion are the most dangerous characteristics of tumor cells and are therefore a major cause of cancer‐related death (Guan, 2015). N-myc downstream-regulated gene 1 (NDRG1) is identified as a metastasis suppressor gene that belongs to the NDRG family, which is comprised of four members: NDRG1‐4 (Keberle, 1964). Reports have documented that NDRG1 localizes to different compartments of the cell, defining its organelle‐specific, pleiotropic functions that affect its interaction with other proteins (Fang et al., 2014). Furthermore, NDRG1 stimulates cell differentiation, growth, stress responses, lipid biosynthesis, and immunity (Kovacevic and Richardson, 2006; Fang et al., 2014), and inhibits cancer progression, metastasis, and angiogenesis (Ellen et al., 2008). Indeed, NDRG1 suppresses metastasis in prostate, breast, colon, and pancreatic cancers; however, it promotes tumor progress in liver, esophagus, cervical, and aggressive triple-negative breast cancer (Menezes et al., 2017; de Nonneville et al., 2022; Villodre et al., 2022). Additionally, NDRG1 contributes to breast cancer aggressiveness by modulating lipid metabolism (Sevinsky et al., 2018). NDRG1 expression is induced under stress conditions involving hypoxia and low levels of iron, thus acting as a stress-responsive protein (Park JS. et al., 2020).

Iron is essential for various biological functions, including DNA and RNA synthesis, DNA repair, metabolism, cell proliferation and differentiation, oxygen transport, and mitochondrial respiration (Sheftel et al., 2012; Zhou et al., 2018). Dysregulation of intracellular iron level is detrimental to cells, as it can induce oxidative stress by producing reactive oxygen species (ROS) *via* the Haber Weiss/Fenton reaction (Renassia and Peyssonnaux, 2019). Therefore, tight regulation of iron uptake, storage, and utilization is necessary to maintain its optimal intracellular level (Jung et al., 2019). Moreover, mitochondria play a crucial role in iron utilization, particularly through the synthesis of iron-containing cofactors - such as iron-sulfur [Fe-S] clusters and heme (Paul et al., 2017).

Unsurprisingly, iron plays a vital role in cancer development, supporting cell growth, metabolism, metastasis, and invasion (Jung et al., 2019). Similarly, a higher incidence of certain cancer types has been described in patients with iron overload (Stevens et al., 1988; Torti and Torti, 2013). Therefore, targeting iron metabolism has been considered a novel therapeutic strategy for cancer.

Numerous studies have shown that iron chelators effectively inhibit tumor growth by depriving cells of iron, resulting in G1/S arrest and the induction of apoptosis (Kovacevic et al., 2011; Li et al., 2012). Recent research has highlighted a potential link between NDRG1 and intracellular iron levels, proposing iron chelators as inhibitors of cell cycle progression and metastasis (Le and Richardson, 2004). Notably, di‐2‐pyridylketone 4,4‐dimethyl-3‐thiosemicarbazone (Dp44mT) and di‐2‐pyridylketone-4‐cyclohexyl‐4‐methyl‐3‐thiosemicarbazone (DpC) have shown higher anti-proliferative and anti-metastatic effects across various cell lines *in vitro* (Park et al., 2020b). These chelators exhibit diverse mechanisms of action including ROS production, upregulation of hypoxia-inducible factor alpha (HIF‐1α) protein signaling, and consequent activation of many genes, one of which is *NDRG1* (Yu et al., 2007; Lane et al., 2014).

Recently, our group has actively contributed to the research on mitochondrial targeting of anti-cancer compounds using delocalized lipophilic phosphonium cations, which has emerged as an effective and specific drug delivery approach (Truksa et al., 2015; Boukalova et al., 2016; Sandoval-Acuna et al., 2016; Rohlenova et al., 2017; Fuentes-Retamal et al., 2020; Sandoval-Acuna et al., 2021; Jadhav et al., 2024). This strategy, initially proposed by Murphy and Smith (2007), involves targeting drugs into mitochondria *via* the triphenyl phosphonium (TPP^+^) moiety, predominantly focusing on inhibitors of mitochondrial respiratory complexes. Notably, these compounds exhibit preferential accumulation in cancer cells due to their higher inner mitochondrial membrane potential (Truksa et al., 2015; Boukalova et al., 2016; Sandoval-Acuna et al., 2016; Rohlenova et al., 2017). We have synthesized and evaluated two mitochondrially targeted iron chelators, mitoDFO and mitoDFX, by conjugating the prototypical iron chelators deferoxamine (DFO) and deferasirox (DFX) to a TPP^+^ moiety, demonstrating their anti-cancer activity in recent studies (Sandoval-Acuna et al., 2021; Jadhav et al., 2024).

To investigate the role of NDRG1 in the response to standard and mitochondrially targeted iron chelators, we tested *NDRG1* knockout (KO) cells and cells overexpressing (OE) three NDRG1 variants listed in the NDRG1 UniProt database entry (Q92597): the full-length (34,945), or two N-terminally truncated isoforms (59,112 and 59,113). Our findings reveal that the function of NDRG1 varies with cellular context. Notably, *NDRG1* KO cells exhibited higher sensitivity to mitoDFX, while overexpression of NDRG1 variants (34945 and 59112) reduced cytotoxicity of mitoDFX. Importantly, full-length NDRG1 overexpression in MDA-MB-231 cells enhanced tumor growth, whereas *NDRG1* knockout and overexpression of the N-terminally truncated NDRG1 isoforms significantly reduced tumor growth.

## 2 Methods

### 2.1 Cell culture

Human cancer cell lines MCF7 (RRID: CVCL_0031), MDA-MB-231 (RRID: CVCL_0062), and non-malignant MRC5 (RRID: CVCL_0440) cells were obtained from the American Type Culture Collection (ATCC). All cells were cultured in a humid incubator at 37°C with 5% CO_2_ in DMEM media (Sigma) supplemented with 10% fetal bovine serum (FBS; Thermo Scientific), 100 U/mL streptomycin/penicillin (Sigma) and 2 mM L-glutamine (PAN Biotech). Additionally, maintenance of overexpressing phenotype was done using Geneticin (250 μg/mL). Cultured cells were authenticated by the STR analysis (Generi Biotech), regularly tested for mycoplasma contamination (MycoAlert Plus detection kit; Lonza) and used within 3 months from thawing.

### 2.2 Generation of NDRG1 knockout (KO) and overexpressing (OE) cell lines

Guide DNAs targeting *NDRG1* exon 4 were generated using the CRISPOR tool (Concordet and Haeussler, 2018) and subcloned into the 55 pX AsCpf1-Venus-NLC crRNA entry vector originating from an AsCpf1-Venus (Bjorn Schuster from IMG, CAS). Obtained plasmids were purified and sequenced. Subsequently, cells were transfected and sorted, and the presence or absence of NDRG1 was evaluated by Western blotting. KO clones were generated first and afterward used for the creation of OE clones. *NDRG1* full-length (CCDS34945.1) and N-terminal truncated variants CCDS59112.1 and CCDS59113.1) were amplified by PCR from cDNA obtained from MCF7 cells treated with Dp44mT and cloned into pcDNA3.4 TOPO vector using BamHI/NotI restriction endonucleases. Primers used for the cloning are shown in the Supplementary Tables S4, S5.

### 2.3 SDS-PAGE and Western blot analysis

Cells were lysed in RIPA buffer containing protease and phosphatase (SERVA) inhibitors. Protein content was determined by the bicinchoninic acid (BCA) method (Thermo Fisher Scientific). Proteins (40 µg) were resolved by 10% SDS-PAGE and transferred to PVDF membrane (Thermo Scientific). Membranes were blocked in 5% milk, washed with tris-buffered saline (TBS) buffer with 0.05% tween and incubated with primary antibodies in 5% bovine serum albumin (Roth) overnight. The next day, membranes were incubated with secondary antibodies, followed by the development of images using chemiluminescent substrates Western Bright™ Sirius (Advansta) or Clarity™ (BioRad) in Azure c600 camera (Azure Biosystems). The list of antibodies used in the study is included in Supplementary Table S1.

### 2.4 Quantitative polymerase chain reaction (qPCR) with reverse transcription

RNA was isolated using RNAzol^®^ RT (Molecular Research Center) according to the instructions of the manufacturer. RNA quantity was measured by using a Nanodrop spectrometer (Thermo Scientific, ND-1000). RNA was then transcribed to cDNA using Revert Aid cDNA First Strand Synthesis Kit (Thermo Fisher Scientific). HOT FIREPol^®^ EvaGreen^®^ qPCR Mix Plus Kit (Solis Biodyne) was then utilized for the quantitative RT-PCR. GenEx software version six was used to analyze the data and was normalized to reference gene for ribosomal protein P0 (*RPLPO*). The sequences and list of primers used for qPCR are shown in Supplementary Table S2.

### 2.5 Reactive oxygen species levels (ROS) and mitochondrial membrane potential

For ROS and mitochondrial membrane potential measurements, 1 × 10^5^ cells were seeded in 12-well plates and incubated overnight. The next day, ROS levels were determined by using the MitoSOX Red dye for mitochondrial superoxide levels (final concentration 2.5 μM; Thermo Fisher Scientific) and 2,7-dichlorodihydrofluorescein-diacetate for cytosolic ROS (DCF-DA; final concentration 5 μM; Sigma-Aldrich). Mitochondrial membrane potential was assessed using tetramethylrhodamine methyl ester (TMRM; final concentration 5 μM; Sigma-Aldrich). Detection of all probes was done on a BD LSRFortessa™ SORP flow cytometer with the following parameters: 488nm_Ex_/585nm_Em_ (DCF-DA), 488nm_Ex_/530nm_Em_ (MitoSOX) and 561nm_Ex_/586nm_Em_ (TMRM). Analysis was done in the FlowJo™ software and results are expressed as a percentage of relative fluorescence units relative to the control.

### 2.6 Measurement of oxygen consumption rate (OCR) and extracellular acidification rate (ECAR)

Cells were seeded on a 96-well plate coated with poly-L-lysine (Sigma-Aldrich). For the measurement of OCR, Mitostress assay was performed using Seahorse XFe96 Analyzer (Agilent Technologies). The assay was run with the following events: oligomycin (1 μM; port A), carbonyl cyanide m‐chlorophenylhydrazone (CCCP; 1 μM; port B), rotenone (1 μM) and antimycin A (1.8 μM; both port C), Hoechst (2 μg/mL; port D). To measure ECAR, the GlycoStress assay was run and measured by the Seahorse XFe96 Analyzer (Agilent Technologies). The assay was run with the following events: glucose (10 mM; port A), oligomycin (1 μM; port B), 2-deoxyglucose (2-DG; 80 mM; port C), and Hoechst (2 μg/mL; port D). For both experiments, cells were counted for normalizations using the ImageXpress Micro XLS analysis system (Molecular Devices). The software Wave was utilized for processing the data.

### 2.7 Confocal microscopy

For confocal imaging, 1 × 10^5^ cells were seeded into a 12-well plate with coverslips. The next day, cells were fixed with 4% paraformaldehyde and washed with phosphate-buffered saline (PBS). Cell permeabilization was done using a permeabilization buffer containing 0.1% Triton X-100 for 10 min. Cells were washed again with PBS, blocked with 5% BSA for 1 h and subsequently incubated with primary NDRG1 antibody for 1 h, washed and followed by incubation with secondary antibody (list included in Supplementary Table S3). Hoechst 33342 (2.5 mg/mL) was added for nuclear staining and incubated for 1 h at room temperature, protected from light. Finally, cells were placed in a microscope glass with 10 μl of mounting media and observed with the Leica SP8 confocal microscope. Fluorescence was detected at 405 nm_Ex_/450 nm_Em_ for Hoechst and 493 nm_Ex_/518 nm_Em_ for the secondary antibody. Images were acquired on Leica SP8 confocal microscopy using a 63x immersion objective. Analysis was performed using the ImageJ software (National Institute of Health).

### 2.8 Real-time cell proliferation and death monitoring

Real-time monitoring was performed using IncuCyte^®^ S3 (Sartorius) in an incubator with standard tissue culture conditions (37°C, 5% CO_2_). 2 × 10^3^ cells were seeded onto a 96-well plate and were treated with mitoDFX (30 nM for MCF7 and 1 µM MDA-MB-231). SYTOX Green (0.5 µM; Thermo Fisher Scientific) was added to detect dead cells. Images were captured every 3 h with two different channels–phase contrast (all cells) and green fluorescence (dead cells) for 72 h. Analysis was performed using the IncuCyte software. All results are shown as normalized confluence/time zero for proliferation and normalized dead counts/phase for cell death measurement. Graphs were generated using Graphpad Prism 9.0 software.

### 2.9 3D invasion assay

MDA-MB-231 and MCF7 cell culture spheroids were formed in 2% agarose microwells (Microtissue^®^; #12–81 large spheroids, Sigma) according to the manufacturer’s protocol. Each spheroid was created from ∼1,500 cells. Spheroids were individually washed with DMEM and embedded in a collagen matrix (1 mg/mL rat tail collagen, 1x DMEM medium, 0.4% NaHCO_3_, 1% FBS and 50 μg/mL gentamicin) overlaid with cultivation medium. The images were acquired using the Leica Thunder system equipped with the LAS-X Navigator software module (Leica) and analyzed using the ImageJ/Fiji software. The spheroid area was delineated using the Threshold or Edge Finder and Binary Mask tool in the ImageJ/Fiji software. The invasion index was calculated as the normalized ratio of the spheroid area of interest to the starting spheroid area.

### 2.10 Animal studies

Athymic Nude mice were subcutaneously injected with 1 × 10^6^ MDA-MB-231 cells. After reaching the volume of 30–50 mm^3^, tumors were scanned twice a week using ultrasound imaging (USI). Tumor size calculations were done with the USI instrument Vevo3100 (VisualSonics) and are presented as relative tumor size. Once the tumor volume reached 1,000 mm^3^ mice were sacrificed and tumors were obtained. Animal ethics was approved by the Czech Academy of Sciences and animal experiments were performed according to the Czech Republic Council guidelines for the Care and Use of Animals in Research and Teaching.

### 2.11 Statistical analysis

All results are expressed as mean ± standard error of the mean (SEM) of at least two or three independent experiments with at least two or more replicates or five different animals. The comparison between experimental groups and control was performed by one-way Analysis of Variance (ANOVA) followed by Dunnett’s multiple comparison or unpaired *t*-test using the GraphPad Prism 9.0 software. Differences were considered significant at *p < 0.05.

## 3 Results

### 3.1 Mitochondrially targeted iron chelators induce NDRG1 expression in breast cancer cells

**FIGURE 1:**
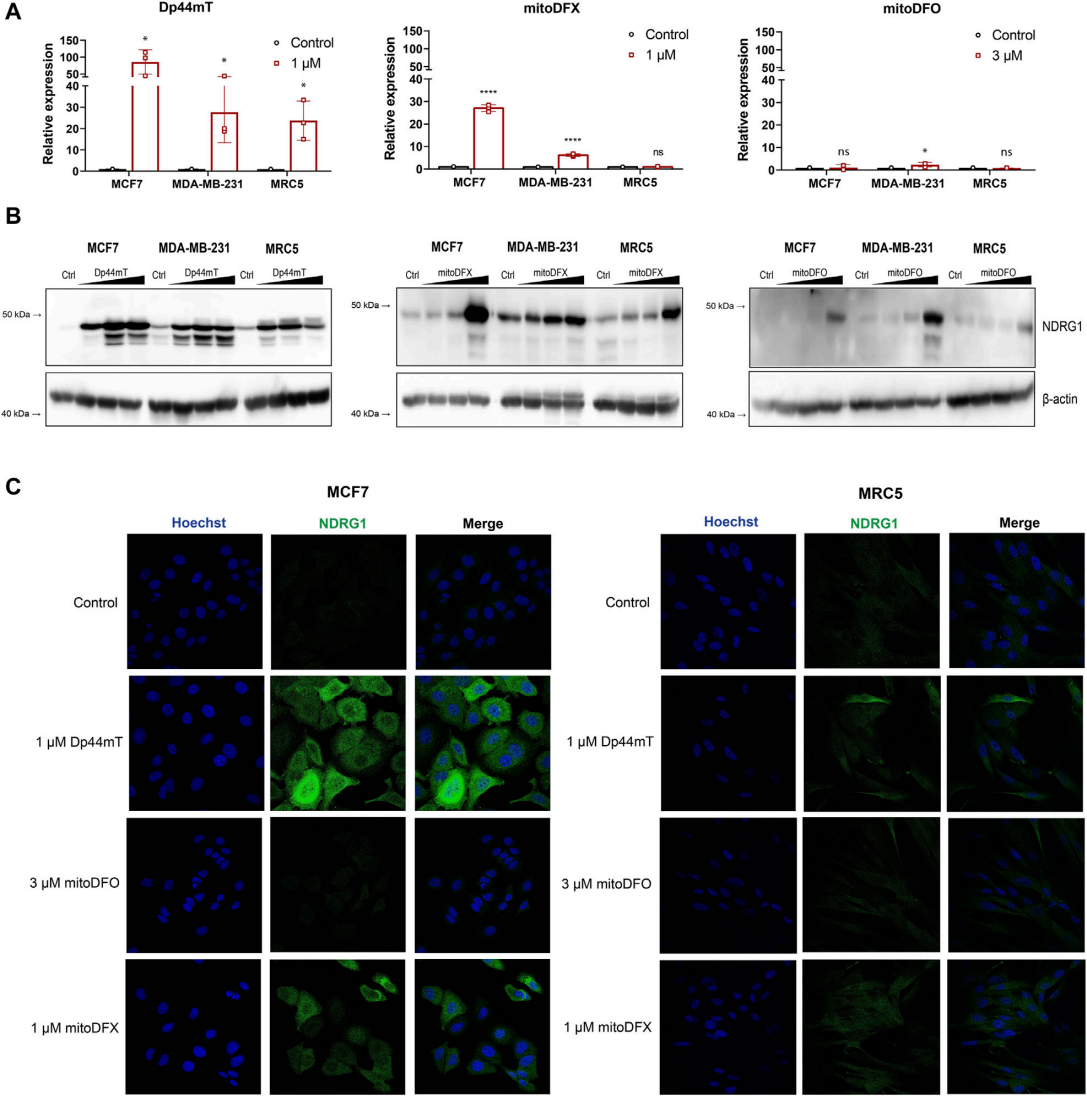
Induction of NDRG1 expression after 24 h exposure to the iron chelator Dp44mT and the mitochondrially targeted iron chelators, mitoDFX and mitoDFO. **(A)** The expression of *NDRG1* in MCF7, MDA-MB-231 and MRC5 cells treated with iron chelators (Dp44mT, mitoDFX and mitoDFO) was analyzed using qPCR. NDRG1 expression was normalized to the human ribosomal large protein P0 gene (*RPLP0*). Data are shown as the relative expression of NDRG1 from three independent experiments with at least three replicates each. **(B)** Western blot images of NDRG1 protein levels were detected in MCF7, MDA-MB-231 and MRC5 cells treated with increasing concentrations of Dp44mT (300 nM, 1 μM and 3 µM), mitoDFX (100 nM, 300 nM and 1 µM) and mitoDFO (300 nM, 1 μM and 3 µM) for 24 h. The Western blot images represent the mean of at least three independent experiments. **(C)** Representative confocal immunofluorescence images were obtained from MCF7 and MDA-MB-231 treated with Dp44mT, mitoDFO and mitoDFX for 24 h and incubated with NDRG1 antibody (Green). Nuclei were stained with Hoechst 33342 (Blue). Scale bars = 20 µm. *p* values were calculated by multiple unpaired *t*-test. *p < 0.05 versus control and ****p < 0.00001.

As previously reported, non-targeted iron chelators, as well as novel thiosemicarbazones, can markedly induce the expression of *NDRG1*, which subsequently suppresses pro-oncogenic mechanisms within cancer cells (Kovacevic et al., 2011; Gutierrez et al., 2014; Menezes et al., 2017; Park et al., 2020b; Chekmarev et al., 2021; Geleta et al., 2021). To fully assess the effect of specific mitochondrial targeting, we employed two compounds introduced by our group-mitochondrially targeted deferoxamine (mDFO) and deferasirox (mitoDFX) (Supplementary Figures S1A, B). Both chelators have shown enhanced anti-tumor efficiency against breast cancer (Sandoval-Acuna et al., 2021; Jadhav et al., 2024), yet, their complete mechanism of action has not been fully determined. We thus wanted to assess whether NDRG1 participates in mediating their effects. An established chelator, Dp44mT, was used as a positive control (Supplementary Figure S1C) and, as expected, it markedly induced the expression of *NDRG1* mRNA in breast cancer cells (MCF7, MDA-MB-231) and non-malignant fibroblasts (MRC5), even though the response was significantly higher in MCF7 cells. Moreover, breast cancer cells treated with mitoDFX demonstrated a significant increase in *NDRG1* mRNA levels with only a mild effect seen on non-malignant MRC5 cells. Similarly, MCF7 and MDA-MB-231 cells treated with mitoDFO showed a slight increase in *NDRG1* mRNA, while MRC5 cells showed no changes in relative *NDRG1* expression (Figure 1A).

We next assessed the total protein level of NDRG1 in malignant and non-malignant cells to study whether NDRG1 protein levels reflect mRNA levels. We observed increased expression of total NDRG1 in a dose-dependent manner with all tested chelators, yet, Dp44mT induced higher NDRG1 levels compared to both mitoDFX and mitoDFO. Moreover, Dp44mT treatment resulted in multiple NDGR1 bands on Western blot, suggesting a possible upregulation of more than one isoform or variant of NDRG1. MitoDFO was the least effective agent as it induced significant changes only at 3 µM concentration (Figure 1B). Subsequently, the levels of NDRG1 protein upon induction were confirmed by confocal microscopy as confocal images showed the identical trend in NDRG1 induction in malignant and non-malignant cells as immunoblotting (Figure 1C). Overall, the results demonstrated that mitochondrially targeted iron chelators mitoDFX and mitoDFO are capable of inducing NDRG1 mRNA and protein levels in breast cancer cells, while only low levels were induced in non-malignant cells. Moreover, it also confirmed that the extent of changes elicited by the mitochondrially targeted iron chelators is lower compared to the changes exhibited by the non-targeted chelator Dp44mT.

### 3.2 Mitochondrially targeted DFO and DFX induce NDRG1 phosphorylation at Ser^330^ and Thr^346^ in breast cancer cells

Previous studies have demonstrated that phosphorylation at two distinct sites, namely, Ser^330^ and Thr^346^, differentially dictates the cellular localization and activity of NDRG1 in various cancer cell types (Park et al., 2018; Sahni et al., 2019). Therefore, we next examined the phosphorylation of NDRG1 at these two sites by immunoblotting in breast cancer and fibroblast cells. Because the mitochondrially targeted iron chelators induced NDRG1 protein level, we tested whether they affected the phosphorylation status of NDRG1 as well. Cells were incubated with Dp44mT (1 μM), mitoDFX (1 and 3 μM), mitoDFO (1 and 3 μM) and with the parental chelators DFO (3 and 100 μM) or DFX (3 and 30 μM) for 24 h.

**FIGURE 2:**
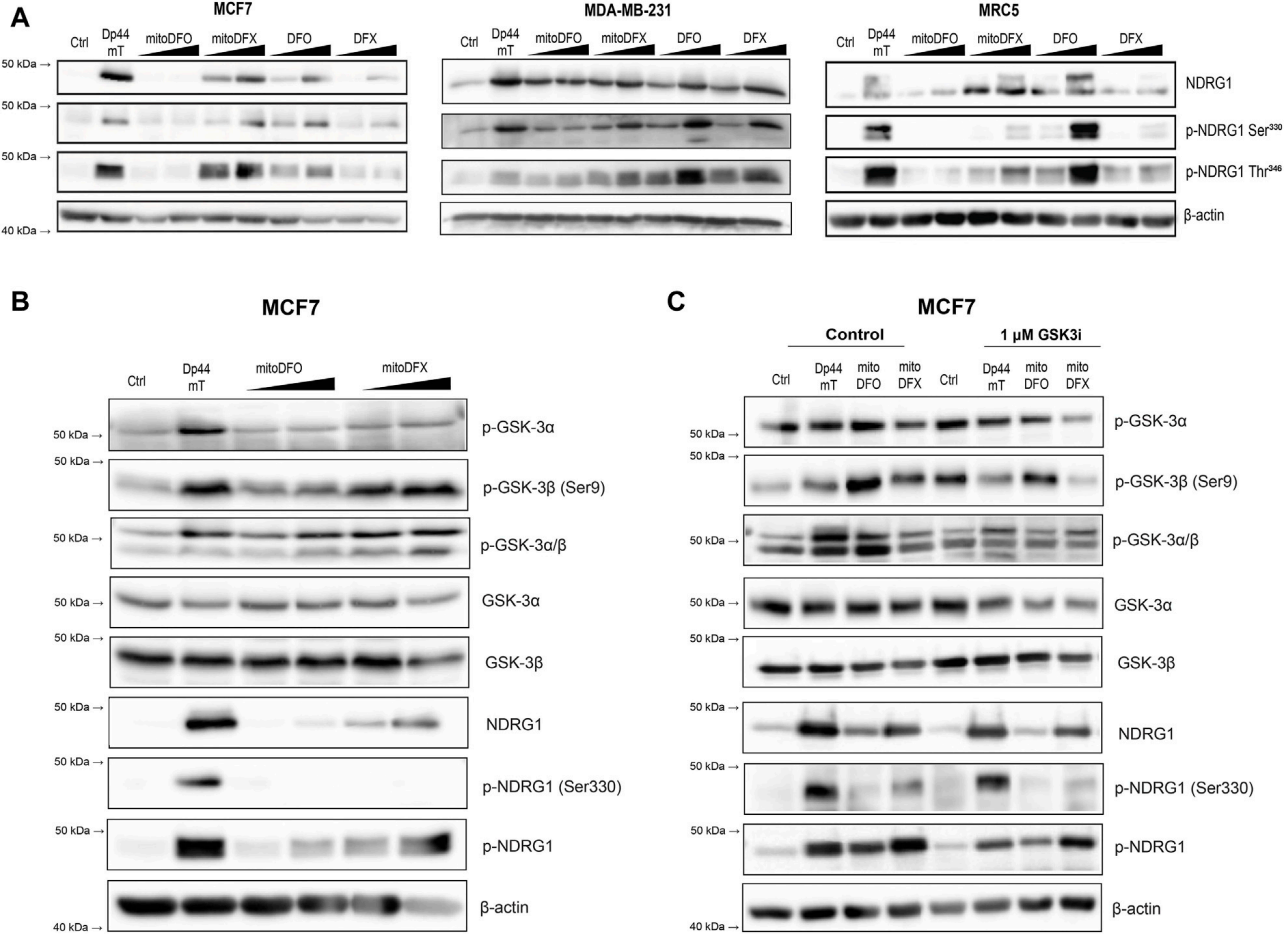
Induction of NDRG1 phosphorylation at Ser^330^ and Thr^346^ after treatment with both conventional and mitochondrially targeted iron chelators observed in breast cancer cells. **(A)** MCF7, MDA-MB-231 and MRC5 cells treated with iron chelators: Dp44mT (1 µM), mitoDFO (1 μM and 3 µM), mitoDFX (1 μM and 3 µM), and their parental compounds: DFO (3 μM and 100 µM), DFX (3 μM and 30 µM) for 24 h. NDRG1 phosphorylation at Ser^330^ and Thr^346^ was detected using specific antibodies, as shown in the figure. **(B)** Western blot images of malignant MCF7 breast cancer cells treated with or without Dp44mT (5 µM), mitoDFO (1 and 3 µM) and mitoDFX (1 and 3 µM) to detect proteins using the antibodies listed in the figure. **(C)** Western blot images of malignant MCF7 breast cancer cells treated with or without Dp44mT (5 µM), mitoDFO (3 µM) and mitoDFX (3 µM) or a GSK3-inhibitor (1 µM) with proteins detected using the antibodies listed in the figure. The Western blot images represent the mean of at least three independent experiments.

Treatment with Dp44mT markedly upregulated both phosphorylated forms of NDRG1 in all cell lines. In contrast, under mitoDFO treatment, there was no alteration in Ser^330^ and only a mild increase in the Thr^346^ phosphorylated form of NDRG1 in both malignant and non-malignant cells. This suggests that targeting DFO into mitochondria reduces its ability to induce NDRG1 phosphorylation as non-targeted DFO was able to significantly induce NDRG1 and its phosphorylated forms in MCF7 as well as MRC5 cells. After treatment with mitoDFX for 24 h, both phosphorylated forms of NDRG1 were significantly increased while non-targeted DFX induced total NDRG1 protein level only slightly and caused a very mild change in both phosphorylated forms of NDRG1, Ser^330^ and Thr^346^, in all tested cell lines (Figure 2A). Therefore, in this case, the mitochondrial targeting resulted in enhanced potency to induce NDRG1 phosphorylation.

Next, we focused on the signaling pathway responsible for the phosphorylation of NDRG1. It is well documented that NDRG1 is phosphorylated at multiple sites by serum- and glucocorticoid-induced kinase 1 (SGK1), which further primes NDRG1 for subsequent phosphorylation by glycogen synthase kinase 3 (GSK3), through which it demonstrates its anti-cancer properties (Murakami et al., 2010). Interestingly, we found that the levels of p-GSK3β and to a lesser extent p-GSK3α were increased upon treatment with Dp44mT and mitoDFX, with mild effect seen with mitoDFO in MCF7 cells (Figure 2B). Furthermore, our results demonstrated that incubation with GSK3 inhibitor reduces the phosphorylation of GSK3β, subsequently resulting in a decrease in p-NDRG1 at Thr^346^, with a mild impact on p-NDRG1 Ser^330^ (Figure 2C). These results suggest the selective phosphorylation of NDRG1 mediated by GSK3α/β after treatment with mitochondrially targeted iron chelators.

### 3.3 Iron chelators specifically induce the expression of NDRG1 but not NDRG2, 3 and 4

**FIGURE 3:**
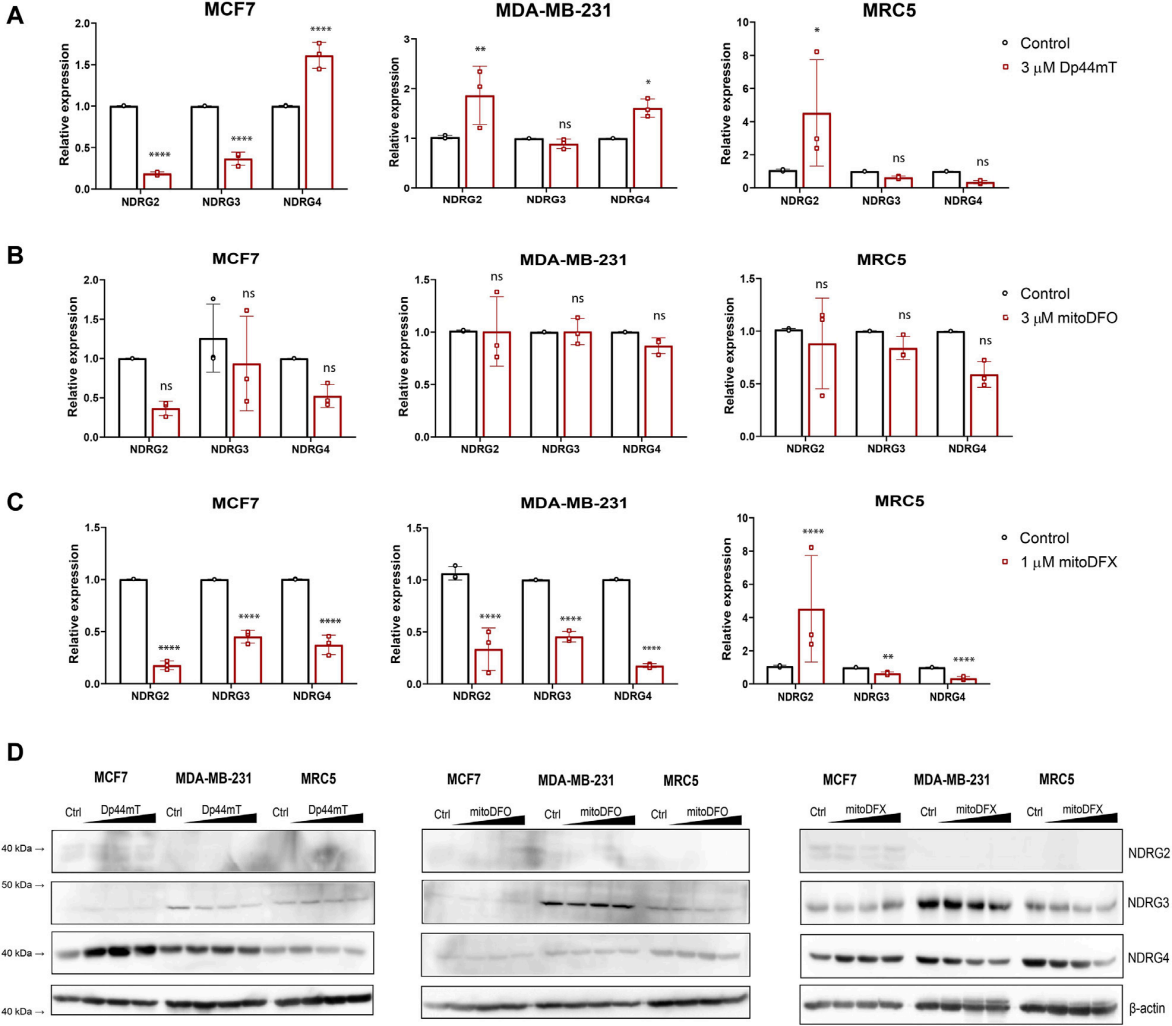
Effects of iron chelators on other members of the NDRG family. **(A–C)** mRNA levels of *NDRG2-4* in breast cancer cells with or without Dp44mt or mitochondrially targeted iron chelators (mitoDFX and mitoDFO) were examined using qPCR and normalized to the human ribosomal large protein P0 gene (*RPLP0*). **(D)** Western blot images of malignant (MCF7 and MDA-MB-231) breast cancer cells and non-malignant (MRC5) cells treated with or without Dp44mT (300 nM, 1 and 3 µM), mitoDFO (300 nM, 1 and 3 µM) and mitoDFX (100 nM, 300 nM and 1 µM) against different family members of NDRG. The Western blot images represent the mean of at least three independent experiments. *p* values were calculated by multiple unpaired *t*-test. *p < 0.05 versus control, **p < 0.001 and ****p < 0.00001.

Several studies have implicated NDRG1 in playing a critical role in iron chelator-mediated cytotoxicity (Lui et al., 2013; Lee et al., 2016; Menezes et al., 2017; Park et al., 2020c; Ito et al., 2020; Geleta et al., 2021). The NDRG family is composed of four members: NDRG1, NDRG2, NDRG3, and NDRG4, which share 53%–65% sequence similarity. However, the role of other family members of NDRG is still not fully understood, with few studies suggesting the involvement of NDRG2 and NDRG3 in carcinogenesis (Wang et al., 2014; Hu et al., 2016; Yu et al., 2019; Zhou et al., 2020). To define whether the other three members of the NDRG family also respond to the treatment with targeted and non-targeted iron chelators, we treated the cells with increasing concentrations of Dp44mT, mitoDFX, and mitoDFO for 24 h Dp44mT slightly increased *NDRG2* and *NDRG4* in MCF7 and MDA-MB-231 cells, with no change or a decrease in other members of the NDRG family (Figure 3A). Treatment with mitoDFO either decreased or did not change the levels of *NDRG2-4* mRNA in the MCF7 and MDA-MB-231 cell lines (Figure 3B). On the other hand, the levels of *NDRG2-4* mRNAs were significantly decreased with mitoDFX treatment in the malignant breast cancer cells (Figure 3C). Furthermore, we performed immunoblotting and observed a lack of upregulation of all tested NDRG members, with slight decrease seen for NDRG3 and 4 upon treatment (Figure 3D). Therefore, this study demonstrates that the upregulating effect in response to iron chelators is specific to NDRG1, with either no change or a slight decrease in other family members.

### 3.4 NDRG1 knockout sensitizes malignant cells to mitoDFX treatment

**FIGURE 4:**
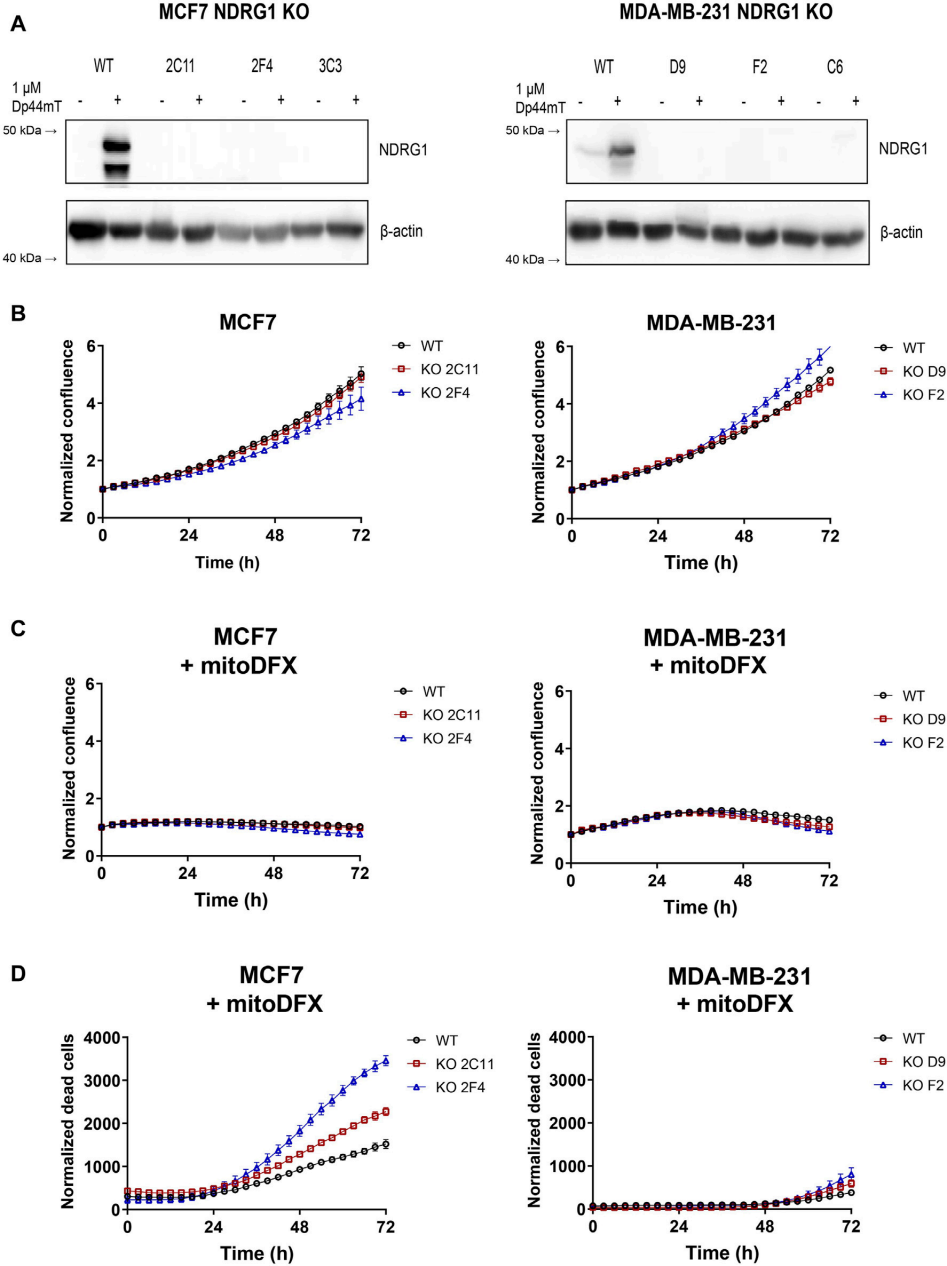
*NDRG1* KO affects the basal growth and response of breast cancers to mitoDFX. **(A)** Western blot images of NDRG1 in MCF7 and MDA-MB-231 *NDRG1* knockout (KO) clones exposed to 1 µM Dp44mT for 24 h to verify successful generation of KO clones. The Dp44mT iron chelator is used as a positive control that induces NDRG1 levels. **(B)** Proliferation curves for MCF7 and MDA-MB-231 wild-type (WT) and KO cells under basal conditions. **(C)** Proliferation curves for MCF7 and MDA-MB-231 WT and KO cells treated for 72 h with 30 nM and 1 µM mitoDFX respectively. The growth curves were monitored using a real-time Incucyte^®^ Sartorius microscope. **(D)** Cell death was measured using Sytox green dye (0.5 µM). **(B–D)** Data are shown as normalized confluence/time zero for proliferation and normalized dead counts/phase for cell death measurement. All values represent the mean ± SEM of three independent experiments with at least three replicates each.

Next, we constructed MCF7 and MDA-MB-231 *NDRG1* knockout (KO) cells to assess the effect of the gene deletion and confirm the role of NDRG1 in the response to iron chelators. The *NDRG1* KO clones were tested using Western blot to verify the deletion of *NDRG1* by treatment with 1 µM Dp44mT. The absence of functional NDRG1 protein in *NDRG1* KO clones was confirmed (Figure 4A), which correlated with the qPCR assay results (Supplementary Figures S2A, B).

Since *NDRG1* is mostly described as a tumor and metastasis suppressor gene, we hypothesized that deletion of the *NDRG1* gene would affect the proliferative rate of breast cancer cell lines. To examine the difference between wild-type (WT) and *NDRG1* KO clones, we used real-time monitoring of cell proliferation using Incucyte^®^ S3 (Sartorius). Two stable clones of each cell type were selected–clones 2C11 and 2F4 for MCF7 and clones D9 and F2 for MDA-MB-231. There was no difference in the basal proliferation rate observed for MCF7 *NDRG1* KO clone 2C11 compared to WT, with only a slight reduction in the 2F4 clone. In MDA-MB-231 cells *NDRG1* KO clone D9 exhibited a basal proliferation rate comparable to WT cells, and there was a slight increase in clone F2 (Figure 4B). We further assessed the response of the *NDRG1* KO clones to mitoDFX and found either no significant change or only slight changes in cytostatic effect in MCF7 and MDA-MB-231 cells compared to WT (Figure 4C). On the other hand, treatment with mitoDFX showed an enhanced cytotoxic effect in MCF7 *NDRG1* KO clones, with only a slightly enhanced cytotoxic effect in MDA-MB-231 *NDRG1* KO clones (Figure 4D). Thus, the results demonstrated enhanced cytotoxic effects of mitoDFX in malignant cells with lack of NDRG1 relative to WT.

### 3.5 Overexpression of full-length NDRG1 (34945) in MCF7 cells reduces the cytotoxic effect of mitoDFX

**FIGURE 5:**
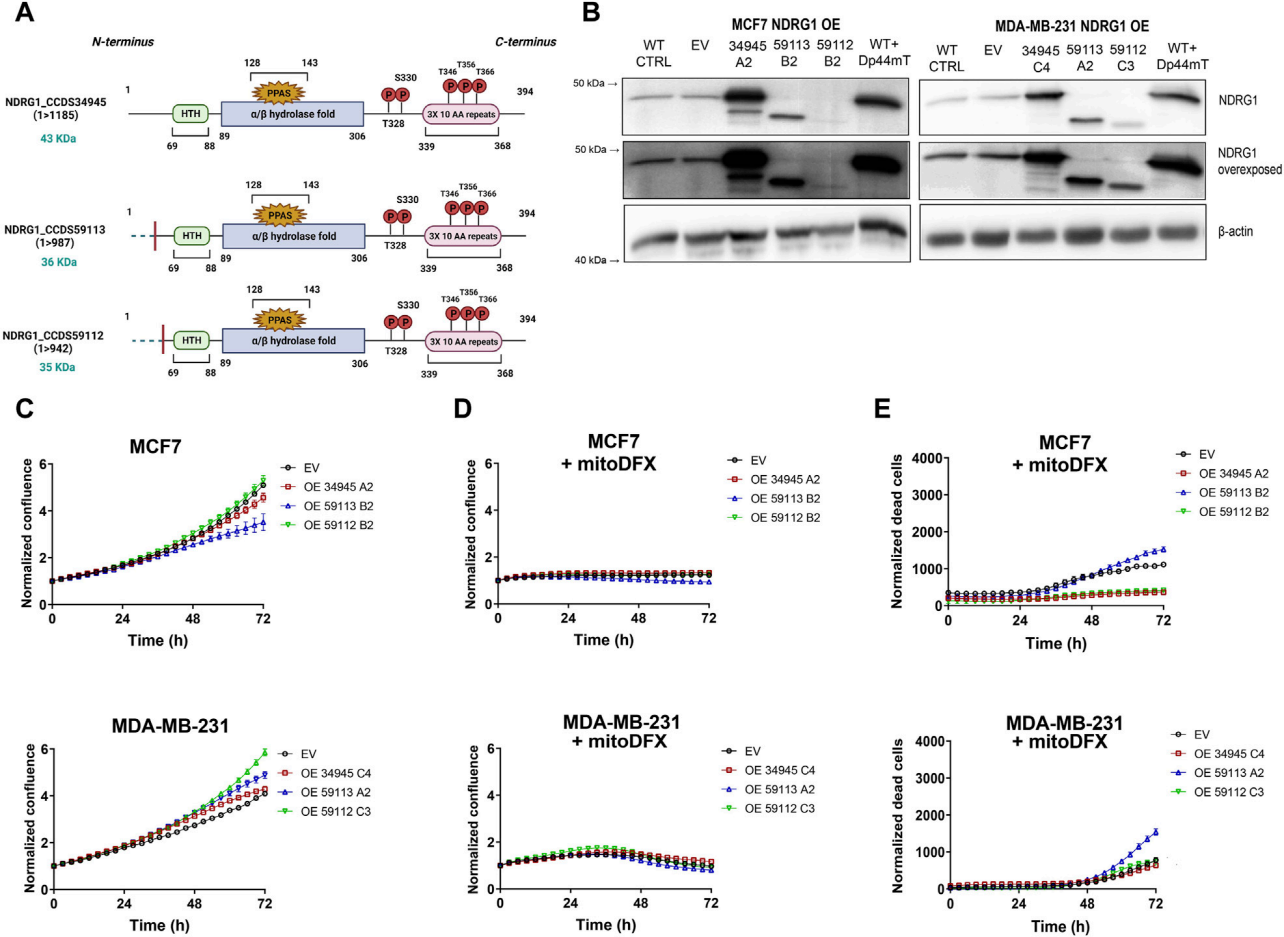
NDRG1 overexpression (OE) affects the basal growth and response of breast cancer cells to mitoDFX. **(A)** Schematic representation of NDRG1 overexpressing isoforms: [full-length (34,945) and two truncated forms (59,113, 59,112)]. **(B)** Western blot images for NDRG1 in MCF7 and MDA-MB-231 *NDRG1* OE clones exposed to 1 µM Dp44mT for 24 h to verify successful generation of OE clones (EV represents empty vector). The Dp44mT iron chelator was used as a positive control that induces NDRG1 levels. **(C)** Proliferation curves for MCF7 and MDA-MB-231 EV and OE clones under basal conditions. The growth curves were monitored using a real-time Incucyte^®^ Sartorius microscope. **(D–E)** Proliferation and cell death curves for MCF7 and MDA-MB-231 EV and OE clones treated with 30 nM and 1 µM mitoDFX respectively. The growth curves were monitored using a real-time Incucyte^®^ Sartorius microscope, and cell death was measured using Sytox green dye (0.5 µM). Data are shown as normalized confluence/time zero for proliferation and normalized dead counts/phase for cell death measurement. All values represent the mean ± SEM of three independent experiments with at least three replicates each.

To determine the role of NDRG1 overexpression in malignant breast cancer cells and its impact on cell proliferation, we generated clones stably overexpressing (OE) full-length *NDRG1* (isoform 34,945) in both malignant cell lines (Figures 5A, B). The successful generation of OE clones of NDRG1 was verified by qPCR results (Supplementary Figures S2A, B) and Western blot (Figure 5B). For the study, WT and empty vector (EV) cells were used as control. To understand the effect of full-length NDRG1 overexpression on cell proliferation, we performed a real-time monitoring assay using Incucyte. Notably, there was no marked difference in the basal proliferative rate of MCF7 or MDA-MB-231 cells after NDRG1 overexpression compared to EV (Figure 5C). We then explored whether the increased levels of full-length NDRG1 modulate the response to iron chelation in breast cancer cells. Our results showed that overexpression of full-length *NDRG1* has no impact on the cytostatic effect of mitoDFX in MCF7 and MDA-MB-231 cells compared to EV (Figure 5D). On the other hand, overexpression of full-length *NDRG1* reduces cytotoxic effects of mitoDFX treatment in MCF7 cells, with no change in MDA-MB-231 cells with full-length NDRG1 OE (Figure 5E).

### 3.6 Overexpression of N-terminally truncated forms of NDRG1 (59113 and 59112) affects the cytotoxic response to mitoDFX in breast cancer cells

We also generated cell lines that overexpressed two N-terminally truncated forms of NDRG1 OE (59113 and 59112) as shown in Figures 5A,B. Furthermore, both the Western blot and qPCR assays verified the successful overexpression of both truncated NDRG1 variants (Supplementary Figures S2A,B). While the truncated version 59112 showed a proliferative rate similar to EV, the 59113 form showed a decrease in basal proliferation rate compared to EV MCF7 cells. On the other hand, both truncated NDRG1 forms exhibited slightly higher basal proliferation rates in MDA-MB-231 breast cancer cells (Figure 5C).

Notably, the cytostatic effect of mitoDFX was similar in EV cells and cells overexpressing truncated NDRG1 versions (Figure 5D). However, treatment with mitoDFX was markedly less cytotoxic in MCF7 cells with the truncated NDRG1 version 59112, showing a similar response to cells carrying the full-length NDRG1. On the other hand, overexpression of the 59113 version of NDRG1 enhanced the cytotoxic response to mitoDFX in MDA-MB-231 cells (Figure 5E).

### 3.7 NDRG1 knockout and overexpression influences oxygen consumption rate, ROS levels and mitochondrial membrane potential

**FIGURE 6:**
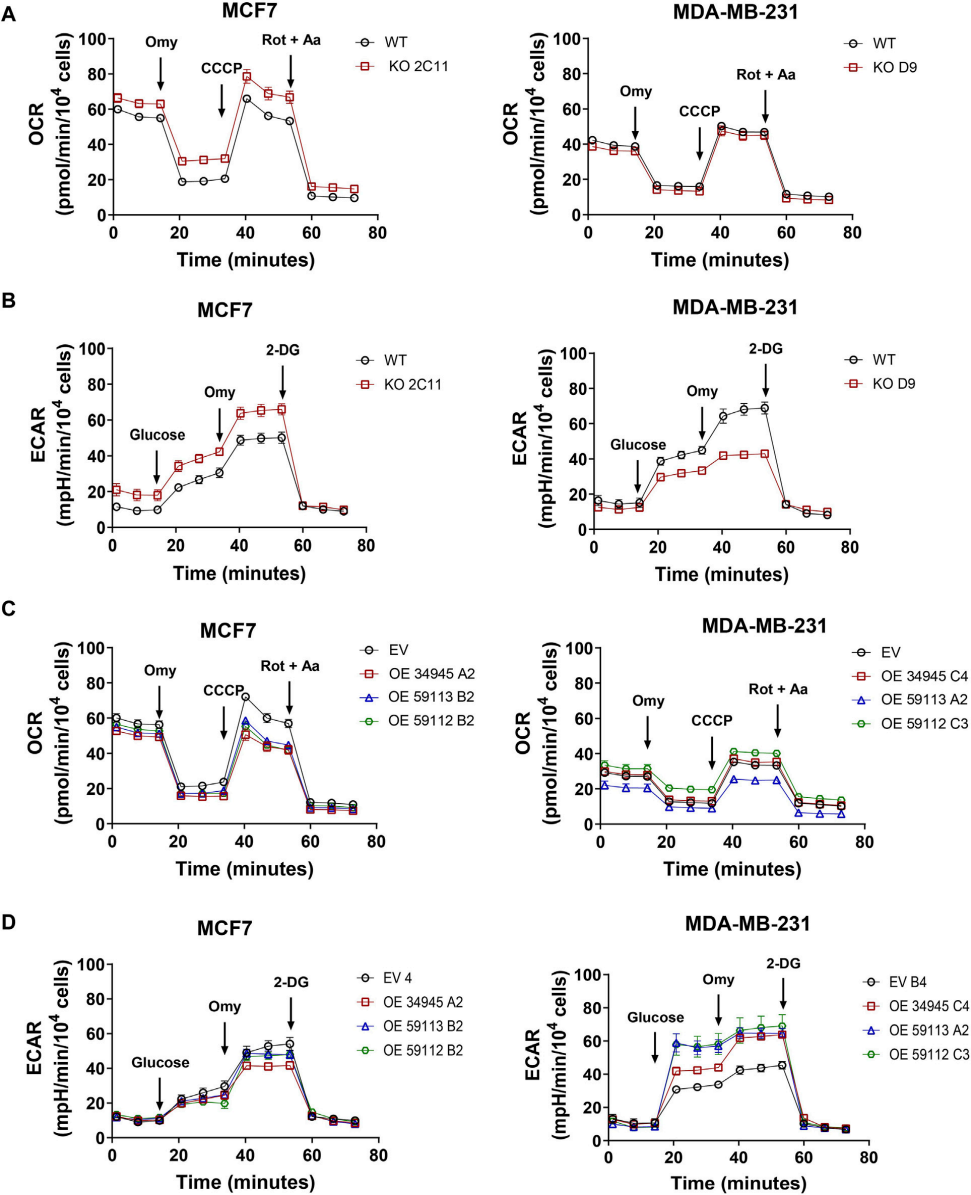
NDRG1 changes cell metabolism in breast cancer cells. **(A)** Profile of oxygen consumption rate (OCR) in MCF7 and MDA-MB-231 wild-type (WT) and *NDRG1* KO clones. **(B)** Glycolysis stress test measuring the extracellular acidification rate (ECAR) in MCF7 and MDA-MB-231 WT and KO clones. **(C)** Profile of OCR in MCF7 and MDA-MB-231 empty vector (EV) and NDRG1 overexpressing clones (OE). **(D)** Glycolysis stress test measuring ECAR in MCF7 and MDA-MB-231 EV and NDRG1 OE clones. OCR was evaluated before and after the addition of oligomycin (Omy, CV inhibitor), CCCP (an uncoupler of OXPHOS), and rotenone plus antimycin A (Rot + AA, CI and CIII inhibitor, respectively). For ECAR, cells were exposed to glucose, oligomycin (Omy) and 2-deoxyglucose (2-DG). All values represent the mean ± SEM of at least three independent experiments with two or more replicates each.

*NDRG1* is a stress-responsive gene induced under hypoxic conditions. Thus, we further investigated the role of NDRG1 expression in oxygen metabolism in cancer cells using a Seahorse XF96 Analyzer. The absence of NDRG1 caused enhanced oxygen consumption rate (OCR) in MCF7, contrary to no significant changes observed in MDA-MB-231 cells (Figure 6A). Additionally, in MCF7 cells, the suppression of NDRG1 enhanced the extracellular acidification rate (ECAR), while significantly reducing ECAR in MDA-MB-231 cells (Figure 6B). On the other hand, overexpression of NDRG1 (full-length and truncated forms) in MCF7 showed lower maximal respiratory capacity compared to EV. Notably, the MDA-MB-231 full-length NDRG1 clone did not show any difference in OCR compared to the EV clone, while the NDRG1 59112 clone demonstrated slightly higher mitochondrial respiration capacity, and the NDRG1 59113 clone demonstrated the lowest OCR (Figure 6C). Likewise, both full-length and truncated forms of NDRG1 showed higher ECAR in MDA-MB-231 with no significant changes observed in MCF7 (Figure 6D).

**FIGURE 7:**
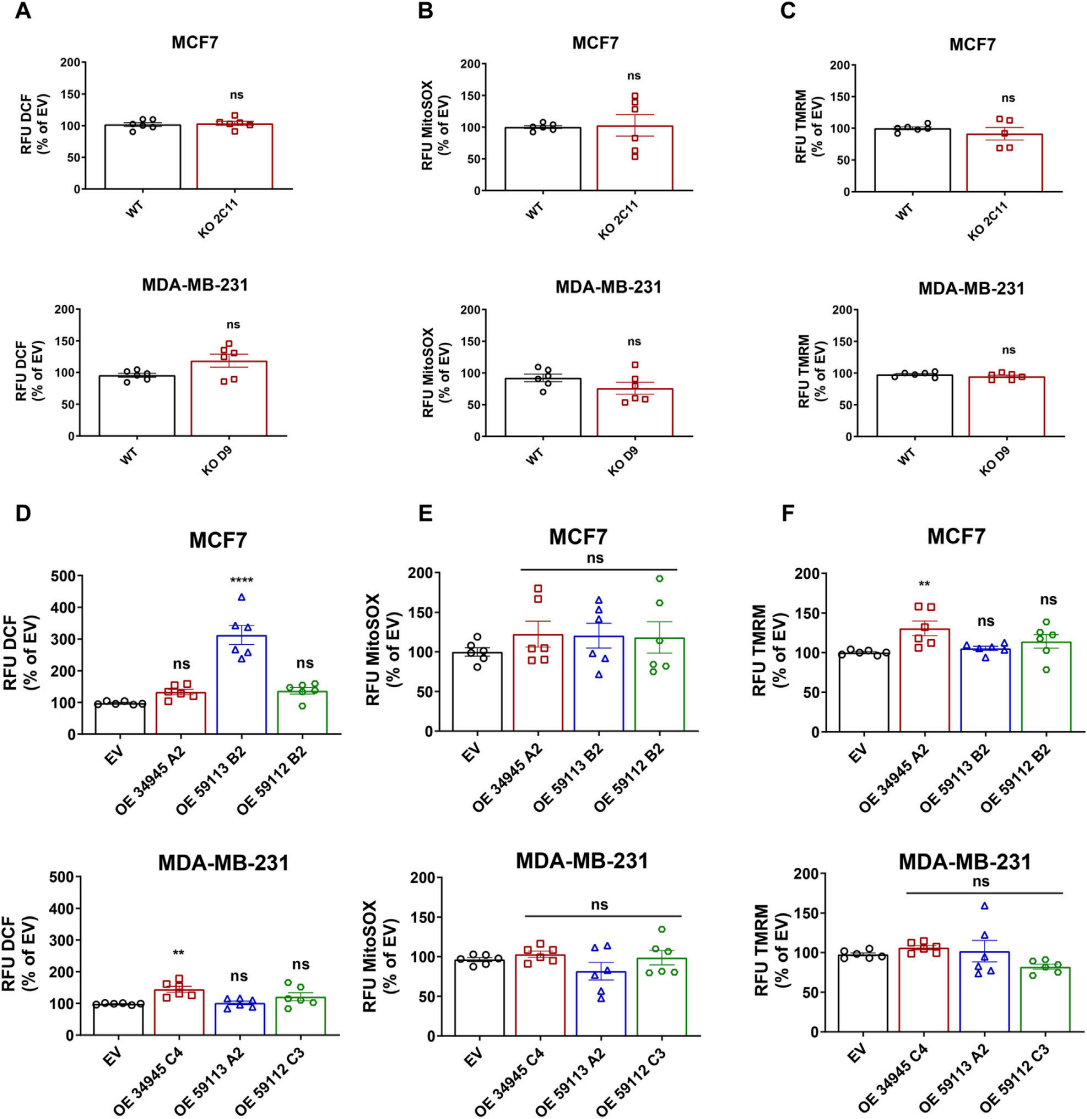
Effect of NDRG1 on ROS levels and mitochondrial membrane potential in NDRG1 KO and OE clones of breast cancer origin. Quantification of DCF-DA fluorescence (cellular ROS; **(A)**), MitoSOX fluorescence (mitochondrial superoxide; **(B)**), and TMRM fluorescence (mitochondrial membrane potential; **(C)**) in MCF7 and MDA-MB-231 wild-type (WT) and NDRG1 knockout (KO) clones. Quantification of DCF-DA fluorescence (cellular ROS; **(D)**), MitoSOX fluorescence (mitochondrial superoxide; **(E)**), and TMRM fluorescence (mitochondrial membrane potential; **(F)**) in MCF7 and MDA-MB-231 WT and NDRG1 OE clones. All values represent the mean ± SEM of at least three independent experiments with two or more replicates each. *p* values were calculated by one-way ANOVA. *p < 0.05 versus control, **p < 0.001, ***p < 0.0001 and ****p < 0.00001.

One of the many functions of NDRG1 is to protect the cell from stress stimuli, such as DNA damage or stress caused by increased levels of reactive oxygen species (ROS). Thus, we further assessed the basal level of ROS to see whether NDRG1 participates in their modulation. To do so, we measured cellular and mitochondrial ROS levels. Notably, none of the *NDRG1* KO clones in either cell line reported any changes in cellular or mitochondrial ROS levels (Figures 7A, B). Despite no changes in KO, follow-up experiments with OE cells showed that both the MCF7 NDRG1 truncated 59113 clone and the MDA-MB-231 NDRG1 full-length OE clone had higher levels of cellular ROS compared to EV control (Figure 7D). However, analysis of mitochondrial ROS production showed no significant change in any NDRG1 OE clone compared to EV cells in any of the cell lines (Figure 7E).

Since mitochondria are the major ROS-producing organelles, and the production can be modified by mitochondrial membrane potential, we evaluated mitochondrial inner membrane potential using the tetramethylrhodamine (TMRM) probe. Loss of NDRG1 showed no significant changes in mitochondrial membrane potential in MCF7 or MDA-MB-231 cells (Figure 7C). On the other hand, overexpression of full-length NDRG1 in MCF7 demonstrated a significant increase in mitochondrial membrane potential, while no changes were seen in MDA-MB-231 cells full-length NDRG1 OE clone (Figure 7F).

### 3.8 NDRG1 affects invasion in MCF7 breast cancer cells *in vitro* and promotes tumor growth of triple-negative breast cancer MDA-MB-231 cells *in vivo*

**FIGURE 8:**
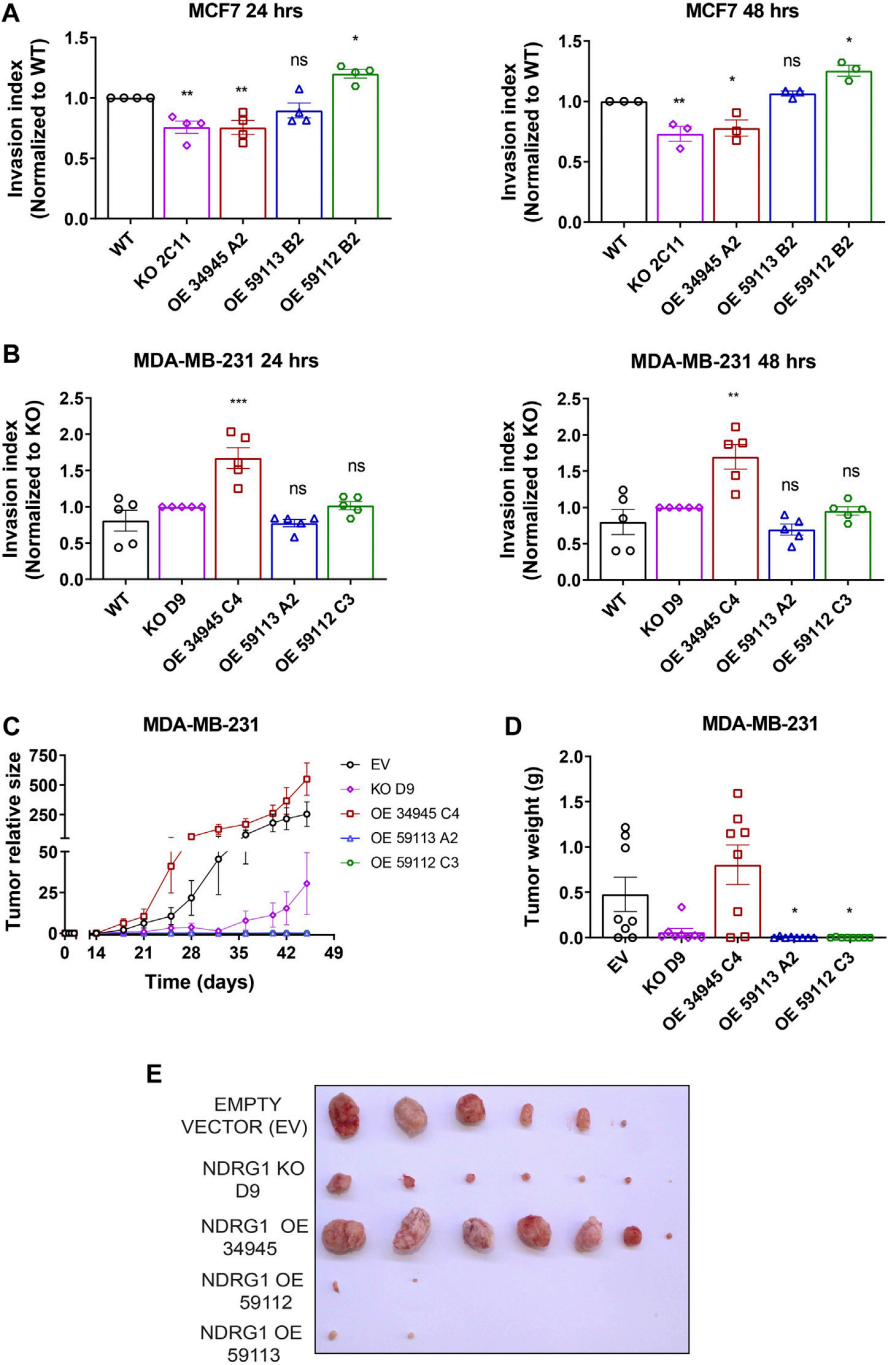
NDRG1 promotes cancer cells invasion *in vitro* and induces tumor growth in triple-negative breast cancer cells *in vivo*. **(A, B)** Quantification of invasion index of MCF7 and MDA-MB-231 cells with their respective *NDRG1* knockout (KO) and overexpressing clones (OE). The invasion index was calculated as the ratio of the area after 24 or 48 h to the initial area (0 h), relative to wild-type (WT) for MCF7 and KO for MDA-MB-231 cells. Data are represented as the mean ± SEM of three independent experiments with at least six replicates each. **(C, D)** Tumor growth curves, tumor weight, and photograph of tumors after dissection of athymic nude mice injected subcutaneously with MDA-MB-231 empty vector (EV), *NDRG1* KO and OE clones (34,945, 59,112 and 59,113). *p* values were calculated by one-way ANOVA. *p < 0.05, **p < 0.001, ***p < 0.0001 versus control.

The most life-threatening aspect of cancer is the development of metastases, which is a major cause of cancer‐related death. Although NDRG1 is mainly recognized as a tumor suppressor, recent studies have also shown it to be a metastatic promoter in a variety of malignancies (de Nonneville et al., 2022; Villodre et al., 2022). Moreover, NDRG1 plays a context-dependent role in cancers by either acting as anti- or pro-oncogenic, depending on the cancer type. Thus, we examined the role of NDRG1 overexpression in influencing the invasion index of the breast cancer cells since tumors become more malignant in hypoxia and NDRG1 expression is dependent on hypoxia. We assessed the ability of modified cells to invade the surrounding collagen matrix when grown as 3D spheres, as already published (Jobe et al., 2018). In MCF7 cells, the overexpression of the truncated 59112 form of *NDRG1* caused significantly enhanced invasion ability of these cells, while *NDRG1* KO and full-length clone showed lower invasive ability with no significant change in 59113 *NDRG1* OE clone (Figure 8A; Supplementary Figure S3A). However, the WT MDA-MB-231 cells and the NDRG1 full-length OE did not form compact spheroids (Supplementary Figure S3B), and we therefore compared the invasion capacity to MDA-MB-231 *NDRG1* KO cells. We found that the invasion capacity of the MDA-MB-231 cells carrying the full-length variant was higher in comparison to *NDRG1* KO and both truncated NDRG1 clones (59113 and 59112) (Figure 8B).

Next, the stable clones of MDA-MB-231 cells carrying either a *NDRG1* KO or plasmids encoding *NDRG1* OE variants (34945, 59112, 59113) or EV were injected into athymic nude mice. Once the tumors were palpable, we monitored them with the USI instrument Vevo3100 (VisualSonics) twice a week and further quantified tumor volume using Vevo Lab 5.6.0 software. The tumors overexpressing full-length NDRG1 grew markedly faster compared to those carrying the EV, while *NDRG1* KO tumors grew significantly less. Importantly, cells carrying both truncated isoforms of NDRG1 had markedly limited tumor initiation and virtually did not grow at all (Figures 8C, D). These data suggest that NDRG1 overexpression promotes invasion and tumor growth in triple-negative breast cancer cells and thus acts as a metastatic driver in a highly aggressive type of breast cancer. Interestingly it appears that this activity requires the N-terminal part of NDRG1 since the truncated isoforms had a very limited ability to form tumors similar to the *NDRG1* KO cells.

## 4 Discussion

Iron is a crucial element required for DNA synthesis and cell growth (Puig et al., 2017). Since cancer cells are highly reliant on iron to maintain their rapid proliferation rate (Basuli et al., 2017), iron chelators have emerged as a potential strategy for cancer treatment. Iron chelators such as deferoxamine (DFO), deferasirox (DFX) and di-2-pyridyl ketone-4,4-dimethyl-3-thiosemicarbazone (Dp44mT) have shown inhibitory effects in several types of cancer *in vitro* and *in vivo* (Merlot et al., 2013). One of the genes induced by iron chelators is N-myc downstream regulated gene (*NDRG1*), which is a well-known metastasis suppressor in various cancer cell types (Le and Richardson, 2004; Kovacevic and Richardson, 2006; Yu et al., 2007; Ellen et al., 2008; Shi et al., 2013; Fang et al., 2014; Lane et al., 2014; Menezes et al., 2017; Park et al., 2020b; Park et al., 2020c; Ito et al., 2020). However, reports have also documented its oncogenic role in aggressive triple-negative breast cancer (TNBC) cells (de Nonneville et al., 2022; Villodre et al., 2022).

Our group has designed and synthesized mitochondrially targeted iron chelators (mitoDFO and mitoDFX), which have shown promising results in cancer cells both *in vitro* and *in vivo* (Sandoval-Acuna et al., 2021; Jadhav et al., 2024). Both iron chelators exert their anticancer effects mainly through depriving cells from biologically active iron and reducing the synthesis of iron-containing cofactors such as iron-sulfur [Fe-S] clusters and heme (Sandoval-Acuna et al., 2021; Jadhav et al., 2024). Additionally, mitoDFX induces lipid peroxidation and glutathione exhaustion, likely due to its redox active nature (Jadhav et al., 2024). For the first time, we have shown that mitochondrially targeted iron chelators, mitoDFO and mitoDFX, upregulate NDRG1 at both mRNA and protein levels in breast cancer cells. Notably, non-targeted iron chelator (Dp44mT) proved to be less selective against non-cancerous cells (MRC5) as compared to targeted iron chelators (mitoDFO and mitoDFX). The differential induction of NDRG1 by targeted and non-targeted iron chelators suggests a potential correlation between NDRG1 expression and the ability of these chelators to induce pseudo-hypoxia, which warrants further investigation.

We noticed that mitoDFO induced NDRG1 protein without affecting its mRNA level, suggesting that posttranslational modifications might control NDRG1 stability. In a recent study by Murakami et al. (2010), NDRG1 phosphorylation at Ser^330^ and Thr^346^ in pancreatic cancer cells was found to be essential for its tumor-suppressive function by inhibiting the nuclear factor kappa B (NF-kB) signaling pathway and CXC chemokine expression (Murakami et al., 2010). Paradoxically, another study demonstrated that NDRG1 phosphorylation at Thr^346^ in hepatocellular carcinoma cells promotes its pro-oncogenic role and showed that phosphorylated NDRG1 (p-NDRG1) at Ser^330^ mainly localizes in the nucleus, while p-NDRG1 at Thr^346^ is predominantly found in the cytoplasm (Park et al., 2018). These findings imply that phosphorylations at two distinct sites, namely, Ser^330^ and Thr^346^, differentially regulate NDRG1 function and its localization. In agreement with these studies, mitochondrially targeted iron chelators (mitoDFO and mitoDFX) enhance p-NDRG1 at Thr^346^ and to a lesser extent Ser^330^, implying the significance of these NDRG1 phosphorylations in the mechanism of action of these agents. Moreover, a correlation between the induction of p-NDRG1 at Thr^346^ and the rise in the total NDRG1 level was observed, which suggests that phosphorylation of NDRG1 induced by mitochondrially targeted iron chelators may be important for the total NDRG1 upregulation.

The phosphorylation of NDRG1 is mediated by either serum/glucocorticoid regulated kinase 1 (SGK1) or glycogen synthase kinase 3 (GSK3), and affects its functionality and subcellular localization (Park et al., 2018). Our work demonstrated that GSK3α/β is involved in the phosphorylation of NDRG1, in accordance with the literature (Ito et al., 2020; Sang et al., 2020). Furthermore, treatment with a GSK3-inhibitor (CHIR99021) reduced NDRG1 phosphorylation at Thr^346^ and it also diminished the level of total NDRG1. These findings suggest that mitochondrially targeted chelators induce NDRG1 not only through transcriptional regulation and induction of “pseudohypoxia”, but also possibly through phosphorylation of NDRG1, which seems to affect its protein level.

We further evaluated the effects of iron chelators on NDRG2, 3, and 4 expressions in breast cancer cells as these members have not been studied as extensively as NDRG1 in the context of carcinogenesis (Ding et al., 2012; Shen et al., 2018; Yu et al., 2019). Although some studies have demonstrated the tumor suppressor role of NDRG2-4, contradictory roles have also been seen in various types of cancer (Liu et al., 2007; Kim et al., 2014; Ren et al., 2014; Wang et al., 2014; Kloten et al., 2016; Jing et al., 2018; Jandrey et al., 2019; Kim et al., 2019; Lee et al., 2021; Zhai et al., 2022). Mitochondrially targeted iron chelators showed specific upregulation of NDRG1. In fact, we demonstrate that treatment with mitochondrially targeted iron chelators leads to reduction in *NDRG2-4* mRNAs, which might suggest pro-oncogenic role of these family members in breast cancers that is inhibited by mitoDFO/mitoDFX. Yet, further studies will be required to delineate the role of other members of NDRG after treatment with mitochondrially targeted iron chelators.

To assess whether NDRG1 is responsible for the anti-cancer effect of the mitochondrially targeted iron chelators and to compare their biological activity, we generated *NDRG1* knockout (KO) and overexpressing (OE) clones (CCDS 34945, CCDS59112 and 59,113) in the MCF7 and MDA-MB-231 cells. Studies have found that NDRG1 modulates proliferation by affecting cyclin D, a key regulator of cell proliferation (Chen et al., 2012). It is notable that *NDRG1* knockout had no effect on breast cancer proliferation. However, NDRG1 overexpression led to reduced proliferation in estrogen receptor-positive (ER+) MCF7 cells, while having the opposite effect in triple-negative breast cancer (TNBC) cells, indicating a cell type-dependent response. A recent study demonstrated the differences between ER + and ER-cell lines might be linked to the presence of the ERα-dependent pathway that mediates downregulation of NDRG1 in ER + breast cell lines, with no such impact seen in TNBC cells (Fotovati et al., 2006; Shehadeh-Tout et al., 2023), which is in agreement with our data. We further report that *NDRG1* KO increased the sensitivity of breast cancer cells to mitoDFX treatment. On the other hand, NDRG1 overexpression of full-length NDRG1 provided resistance in ER + breast cancer cells against mitoDFX, which suggests that high expression of NDRG1 might modulate response to the treatment and cause resistance. Interestingly, the cytostatic effect of mitoDFX reflected in the inhibition of cell proliferation, was unaffected by either overexpression or knockout of NDRG1. This could be possibly due to additional factors besides NDRG1 at play in suppressing proliferation upon iron depletion (Yu et al., 2007).

A recent study showed the unique role of NDRG1 in modulating glycolysis of pancreatic ductal adenocarcinoma cancer cells, resulting in inhibited cell growth (Liu W. et al., 2017). In line with these findings, *NDRG1* KO resulted in enhanced glycolysis, implying that NDRG1 suppresses glycolysis and lactic acid production, which is coupled with reduced cell proliferation in ER + breast cancer cells. On the other hand, NDRG1 overexpression induced glycolysis with concurrent decrease in mitochondrial respiration, facilitating rapid cell proliferation, which is in consistent with the enhanced proliferation rate observed in TNBC cells. Indeed, further studies are needed to elucidate the underlying mechanisms through which NDRG1 exerts its metabolic switch activity in breast cancer.

*NDRG1* is a stress response gene highly upregulated by hypoxia and is responsible for resistance to chemotherapy (Guo et al., 2020). It is frequently characterized as a tumor suppressor by inhibiting the migration and invasion of cancer cells (Bandyopadhyay et al., 2004; Liu et al., 2012). In the current study, we showed that overexpression of full-length NDRG1 suppressed invasion in ER+ breast cancer. However, overexpression of full-length NDRG1 functions as a promoter of tumor growth and metastasis in ER-, aggressive breast cancers, which is consistent with the previous work demonstrating where silencing of *NDRG1* prevented tumor formation in highly aggressive TNBC (Villodre et al., 2020; Villodre et al., 2022; Joshi et al., 2024). Thus, these results highlight induced NDRG1 expression as a common feature of poor prognosis in TNBC, with its elevated expression strongly correlated with an aggressive metabolic shift towards glycolysis and its impact on lipid metabolism to promote growth and metastasis (Liu Q. et al., 2017; Sevinsky et al., 2018).

A recent investigation demonstrated that the truncated NDRG1 isoform was observed only in prostate cancer cells and not in normal prostate epithelial cells, suggesting a pro-oncogenic role of NDRG1 (Ghalayini et al., 2013). Furthermore, NDRG1 has been shown to localize in the nucleus following treatment with iron chelators and DNA-damaging agents, potentially conferring resistance to chemotherapy, which was further supported by the importance of the N-terminus for nuclear localization (Kurdistani et al., 1998; Park et al., 2018). Paradoxically, a recent study found that exposure to hypoxia-triggered nuclear localization via phosphopantetheine attachment site (PPAS); however, the deletion of the N-terminus region of NDRG1 did not influence its cellular localization (Shi et al., 2013). Therefore, to investigate the role of NDRG1 isoforms, we generated N-terminally truncated isoforms (59,112 and 59,113). Interestingly, we observed high mRNA but relatively low protein levels in N-terminally truncated NDRG1 variants, suggesting that the N-terminal region might regulate the stability of NDRG1. Importantly, both N-terminally truncated NDRG1 isoforms prevented limited tumor growth, highlighting the critical role of the N-terminal region in its oncogenic function, potentially affecting NDRG1 interactions or stability.

In summary, we have demonstrated that mitochondrially targeted iron chelators, mitoDFO and mitoDFX, upregulate NDRG1 expression and induce Thr^346^ phosphorylation *via* the GSK3α/β kinase in breast cancer cells. Moreover, *NDRG1* KO and OE modulates the sensitivity/resistance to mitoDFX treatment. Our work also highlights a novel facet of NDRG1 in modulating glycolytic and mitochondrial respiration of breast cancer cells. Finally, our results support the oncogenic properties of NDRG1 in TNBC and show the importance of the N-terminal region of NDRG1 in tumor initiation, growth and invasion in aggressive breast cancer. Thus, these results suggest that NDRG1 may serve as a promising therapeutic target for anti-metastasis and targeted therapies in TNBC.

## Data Availability

The raw data supporting the conclusion of this article will be made available by the authors, without undue reservation.

## Supplementary material

SUPPLEMENTARY FIGURE S1 Chemical structures of: **(A)** mitoDFO, **(B)** mitoDFX, and **(C)** Dp44mT.

SUPPLEMENTARY FIGURE S2 Verification of NDRG1 KO and OE clones in breast cancer cells and the effect of NDRG1 on invasion measured by 3D spheroid generation. **(A, B)** mRNA levels of *NDRG1* in MCF7 and MDA-MB-231 *NDRG1* KO and OE clones exposed to 1 µM mitoDFX for 24 h to verify successful generation of KO and OE clones. Data are normalized to the human ribosomal large protein P0 gene (RPLP0). All data are shown as the relative expression of NDRG1 from three independent experiments with at least three replicates each. *p* values were calculated by multiple unpaired *t*-test. ^*^ *p* < 0.05 versus control, **p< 0.001 and *****p* < 0.00001.

SUPPLEMENTARY FIGURE S3 The effect of NDRG1 on invasion was measured by 3D spheroid generation. **(A, B)** Generation of spheroids from MCF7 and MDA-MB-231 breast cancer cells carrying either *NDRG1* knockout (KO) or overexpressing clones (both full-length and truncated versions) visualized and imaged at different time-points (0, 24 and 48 h) to determine the invasion ability in 3D environment.

## References

[B1] Bandyopadhyay S. Pai S. K. Hirota S. Hosobe S. Takano Y. Saito K. (2004). Role of the putative tumor metastasis suppressor gene Drg-1 in breast cancer progression. Oncogene 23 (33), 5675–5681. 10.1038/sj.onc.1207734 15184886

[B2] Basuli D. Tesfay L. Deng Z. Paul B. Yamamoto Y. Ning G. (2017). Iron addiction: a novel therapeutic target in ovarian cancer. Oncogene 36 (29), 4089–4099. 10.1038/onc.2017.11 28319068 PMC5540148

[B3] Boukalova S. Stursa J. Werner L. Ezrova Z. Cerny J. Bezawork-Geleta A. (2016). Mitochondrial targeting of metformin enhances its activity against pancreatic cancer. Mol. Cancer Ther. 15 (12), 2875–2886. 10.1158/1535-7163.MCT-15-1021 27765848

[B4] Chekmarev J. Azad M. G. Richardson D. R. (2021). The oncogenic signaling disruptor, NDRG1: molecular and cellular mechanisms of activity. Cells 10 (9), 2382. 10.3390/cells10092382 34572031 PMC8465210

[B5] Chen Z. Zhang D. Yue F. Zheng M. Kovacevic Z. Richardson D. R. (2012). The iron chelators Dp44mT and DFO inhibit TGF-β-induced epithelial-mesenchymal transition via up-regulation of N-Myc downstream-regulated gene 1 (NDRG1). J. Biol. Chem. 287 (21), 17016–17028. 10.1074/jbc.M112.350470 22453918 PMC3366822

[B6] Concordet J. P. Haeussler M. (2018). CRISPOR: intuitive guide selection for CRISPR/Cas9 genome editing experiments and screens. Nucleic Acids Res. 46 (W1), W242-W245–W5. 10.1093/nar/gky354 29762716 PMC6030908

[B7] de Nonneville A. Finetti P. Mamessier E. Bertucci F. (2022). RE: NDRG1 in aggressive breast cancer progression and brain metastasis. J. Natl. Cancer Inst. 114 (7), 1046–1047. 10.1093/jnci/djac031 35148398 PMC9275762

[B8] Ding W. Zhang J. Yoon J. G. Shi D. Foltz G. Lin B. (2012). NDRG4 is downregulated in glioblastoma and inhibits cell proliferation. OMICS 16 (5), 263–267. 10.1089/omi.2011.0146 22489821 PMC3339384

[B9] Ellen T. P. Ke Q. Zhang P. Costa M. (2008). NDRG1, a growth and cancer related gene: regulation of gene expression and function in normal and disease states. Carcinogenesis 29 (1), 2–8. 10.1093/carcin/bgm200 17916902

[B10] Fang B. A. Kovacevic Z. Park K. C. Kalinowski D. S. Jansson P. J. Lane D. J. (2014). Molecular functions of the iron-regulated metastasis suppressor, NDRG1, and its potential as a molecular target for cancer therapy. Biochim. Biophys. Acta 1845 (1), 1–19. 10.1016/j.bbcan.2013.11.002 24269900

[B11] Fotovati A. Fujii T. Yamaguchi M. Kage M. Shirouzu K. Oie S. (2006). 17Beta-estradiol induces down-regulation of Cap43/NDRG1/Drg-1, a putative differentiation-related and metastasis suppressor gene, in human breast cancer cells. Clin. Cancer Res. 12 (10), 3010–3018. 10.1158/1078-0432.CCR-05-1962 16707596

[B12] Fuentes-Retamal S. Sandoval-Acuna C. Peredo-Silva L. Guzman-Rivera D. Pavani M. Torrealba N. (2020). Complex mitochondrial dysfunction induced by TPP(+)-Gentisic acid and mitochondrial translation inhibition by doxycycline evokes synergistic lethality in breast cancer cells. Cells 9 (2), 407. 10.3390/cells9020407 32053908 PMC7072465

[B13] Geleta B. Park K. C. Jansson P. J. Sahni S. Maleki S. Xu Z. (2021). Breaking the cycle: targeting of NDRG1 to inhibit bi-directional oncogenic cross-talk between pancreatic cancer and stroma. FASEB J. 35 (2), e21347. 10.1096/fj.202002279R 33484481

[B14] Ghalayini M. K. Dong Q. Richardson D. R. Assinder S. J. (2013). Proteolytic cleavage and truncation of NDRG1 in human prostate cancer cells, but not normal prostate epithelial cells. Biosci. Rep. 33 (3), e00042. 10.1042/BSR20130042 23634903 PMC3679596

[B15] Guan X. (2015). Cancer metastases: challenges and opportunities. Acta Pharm. Sin. B 5 (5), 402–418. 10.1016/j.apsb.2015.07.005 26579471 PMC4629446

[B16] Guo D. D. Xie K. F. Luo X. J. (2020). Hypoxia-induced elevated NDRG1 mediates apoptosis through reprograming mitochondrial fission in HCC. Gene 741, 144552. 10.1016/j.gene.2020.144552 32165297

[B17] Gutierrez E. Richardson D. R. Jansson P. J. (2014). The anticancer agent di-2-pyridylketone 4,4-dimethyl-3-thiosemicarbazone (Dp44mT) overcomes prosurvival autophagy by two mechanisms: persistent induction of autophagosome synthesis and impairment of lysosomal integrity. J. Biol. Chem. 289 (48), 33568–33589. 10.1074/jbc.M114.599480 25301941 PMC4246109

[B18] Hu W. Fan C. Jiang P. Ma Z. Yan X. Di S. (2016). Emerging role of N-myc downstream-regulated gene 2 (NDRG2) in cancer. Oncotarget 7 (1), 209–223. 10.18632/oncotarget.6228 26506239 PMC4807993

[B19] Ito H. Watari K. Shibata T. Miyamoto T. Murakami Y. Nakahara Y. (2020). Bidirectional regulation between NDRG1 and GSK3β controls tumor growth and is targeted by differentiation inducing factor-1 in glioblastoma. Cancer Res. 80 (2), 234–248. 10.1158/0008-5472.CAN-19-0438 31723002

[B21] Jadhav S. B. Sandoval Acuña C. Pacior Pampin Y. Klanicova K. Blazkova K. Sedlacek R. (2024). Mitochondrially targeted deferasirox kills cancer cells via simultaneous iron deprivation and ferroptosis induction. bioRxiv. 2024, 575692. 10.1101/2024.01.17.575692

[B22] Jandrey E. H. F. Moura R. P. Andrade L. N. S. Machado C. L. Campesato L. F. Leite K. R. M. (2019). NDRG4 promoter hypermethylation is a mechanistic biomarker associated with metastatic progression in breast cancer patients. NPJ Breast Cancer 5, 11. 10.1038/s41523-019-0106-x 30963110 PMC6450950

[B23] Jing J. S. Li H. Wang S. C. Ma J. M. Yu L. Q. Zhou H. (2018). NDRG3 overexpression is associated with a poor prognosis in patients with hepatocellular carcinoma. Biosci. Rep. 38 (6). 10.1042/BSR20180907 PMC6435526 30413609

[B24] Jobe N. P. Zivicova V. Mifkova A. Rosel D. Dvorankova B. Kodet O. (2018). Fibroblasts potentiate melanoma cells *in vitro* invasiveness induced by UV-irradiated keratinocytes. Histochem Cell. Biol. 149 (5), 503–516. 10.1007/s00418-018-1650-4 29435761

[B25] Joshi V. Stacey A. Feng Y. F. Kalita-de Croft P. Duijf P. H. G. Simpson P. T. (2024). NDRG1 is a prognostic biomarker in breast cancer and breast cancer brain metastasis. J. Pathol. Clin. Res. 10 (2). 10.1002/2056-4538.12364 PMC10839626 40530439

[B26] Jung M. Mertens C. Tomat E. Brune B. (2019). Iron as a central player and promising target in cancer progression. Int. J. Mol. Sci. 20 (2), 273. 10.3390/ijms20020273 30641920 PMC6359419

[B27] Keberle H. (1964). The biochemistry of desferrioxamine and its relation to iron metabolism. Ann. N. Y. Acad. Sci. 119, 758–768. 10.1111/j.1749-6632.1965.tb54077.x 14219455

[B28] Kim M. C. Park M. H. Kang S. H. Bae Y. K. (2019). NDRG3 protein expression is associated with aggressive biologic phenotype and unfavorable outcome in patients with invasive breast cancer. Int. J. Clin. Exp. Pathol. 12 (10), 3886–3893. 31933778 PMC6949768

[B29] Kim M. J. Lim J. Yang Y. Lee M. S. Lim J. S. (2014). N-myc downstream-regulated gene 2 (NDRG2) suppresses the epithelial-mesenchymal transition (EMT) in breast cancer cells via STAT3/Snail signaling. Cancer Lett. 354 (1), 33–42. 10.1016/j.canlet.2014.06.023 25153349

[B30] Kloten V. Schlensog M. Eschenbruch J. Gasthaus J. Tiedemann J. Mijnes J. (2016). Abundant NDRG2 expression is associated with aggressiveness and unfavorable patients' outcome in basal-like breast cancer. PLoS One 11 (7), e0159073. 10.1371/journal.pone.0159073 27400234 PMC4939972

[B31] Kovacevic Z. Richardson D. R. (2006). The metastasis suppressor, Ndrg-1: a new ally in the fight against cancer. Carcinogenesis 27 (12), 2355–2366. 10.1093/carcin/bgl146 16920733

[B32] Kovacevic Z. Sivagurunathan S. Mangs H. Chikhani S. Zhang D. Richardson D. R. (2011). The metastasis suppressor, N-myc downstream regulated gene 1 (NDRG1), upregulates p21 via p53-independent mechanisms. Carcinogenesis 32 (5), 732–740. 10.1093/carcin/bgr046 21398495

[B33] Kurdistani S. K. Arizti P. Reimer C. L. Sugrue M. M. Aaronson S. A. Lee S. W. (1998). Inhibition of tumor cell growth by RTP/rit42 and its responsiveness to p53 and DNA damage. Cancer Res. 58 (19), 4439–4444. 9766676

[B34] Lane D. J. Mills T. M. Shafie N. H. Merlot A. M. Saleh Moussa R. Kalinowski D. S. (2014). Expanding horizons in iron chelation and the treatment of cancer: role of iron in the regulation of ER stress and the epithelial-mesenchymal transition. Biochim. Biophys. Acta 1845 (2), 166–181. 10.1016/j.bbcan.2014.01.005 24472573

[B35] Le N. T. Richardson D. R. (2004). Iron chelators with high antiproliferative activity up-regulate the expression of a growth inhibitory and metastasis suppressor gene: a link between iron metabolism and proliferation. Blood 104 (9), 2967–2975. 10.1182/blood-2004-05-1866 15251988

[B36] Lee A. Lim S. Oh J. Lim J. Yang Y. Lee M. S. (2021). NDRG2 expression in breast cancer cells downregulates PD-L1 expression and restores T cell proliferation in tumor-coculture. Cancers (Basel) 13 (23), 6112. 10.3390/cancers13236112 34885221 PMC8656534

[B37] Lee J. C. Chiang K. C. Feng T. H. Chen Y. J. Chuang S. T. Tsui K. H. (2016). The iron chelator, Dp44mT, effectively inhibits human oral squamous cell carcinoma cell growth *in vitro* and *in vivo* . Int. J. Mol. Sci. 17 (9), 1435. 10.3390/ijms17091435 27589737 PMC5037714

[B38] Li J. Liu X. Wang H. Zhang W. Chan D. C. Shi Y. (2012). Lysocardiolipin acyltransferase 1 (ALCAT1) controls mitochondrial DNA fidelity and biogenesis through modulation of MFN2 expression. Proc. Natl. Acad. Sci. U. S. A. 109 (18), 6975–6980. 10.1073/pnas.1120043109 22509026 PMC3345005

[B39] Liu N. Wang L. Liu X. Yang Q. Zhang J. Zhang W. (2007). Promoter methylation, mutation, and genomic deletion are involved in the decreased NDRG2 expression levels in several cancer cell lines. Biochem. Biophys. Res. Commun. 358 (1), 164–169. 10.1016/j.bbrc.2007.04.089 17470364

[B40] Liu Q. Luo Q. Halim A. Song G. (2017b). Targeting lipid metabolism of cancer cells: a promising therapeutic strategy for cancer. Cancer Lett. 401, 39–45. 10.1016/j.canlet.2017.05.002 28527945

[B41] Liu W. Xing F. Iiizumi-Gairani M. Okuda H. Watabe M. Pai S. K. (2012). N-myc downstream regulated gene 1 modulates Wnt-β-catenin signalling and pleiotropically suppresses metastasis. EMBO Mol. Med. 4 (2), 93–108. 10.1002/emmm.201100190 22246988 PMC3306556

[B42] Liu W. Zhang B. Hu Q. Qin Y. Xu W. Shi S. (2017a). A new facet of NDRG1 in pancreatic ductal adenocarcinoma: suppression of glycolytic metabolism. Int. J. Oncol. 50 (5), 1792–1800. 10.3892/ijo.2017.3938 28350132

[B43] Lui G. Y. Obeidy P. Ford S. J. Tselepis C. Sharp D. M. Jansson P. J. (2013). The iron chelator, deferasirox, as a novel strategy for cancer treatment: oral activity against human lung tumor xenografts and molecular mechanism of action. Mol. Pharmacol. 83 (1), 179–190. 10.1124/mol.112.081893 23074173

[B44] Menezes S. V. Sahni S. Kovacevic Z. Richardson D. R. (2017). Interplay of the iron-regulated metastasis suppressor NDRG1 with epidermal growth factor receptor (EGFR) and oncogenic signaling. J. Biol. Chem. 292 (31), 12772–12782. 10.1074/jbc.R117.776393 28615452 PMC5546018

[B45] Merlot A. M. Kalinowski D. S. Richardson D. R. (2013). Novel chelators for cancer treatment: where are we now? Antioxid. Redox Signal 18 (8), 973–1006. 10.1089/ars.2012.4540 22424293

[B46] Murakami Y. Hosoi F. Izumi H. Maruyama Y. Ureshino H. Watari K. (2010). Identification of sites subjected to serine/threonine phosphorylation by SGK1 affecting N-myc downstream-regulated gene 1 (NDRG1)/Cap43-dependent suppression of angiogenic CXC chemokine expression in human pancreatic cancer cells. Biochem. Biophys. Res. Commun. 396 (2), 376–381. 10.1016/j.bbrc.2010.04.100 20416281

[B47] Murphy M. P. Smith R. A. (2007). Targeting antioxidants to mitochondria by conjugation to lipophilic cations. Annu. Rev. Pharmacol. Toxicol. 47, 629–656. 10.1146/annurev.pharmtox.47.120505.105110 17014364

[B48] Park J. S. Burckhardt C. J. Lazcano R. Solis L. M. Isogai T. Li L. (2020a). Mechanical regulation of glycolysis via cytoskeleton architecture. Nature 578 (7796), 621–626. 10.1038/s41586-020-1998-1 32051585 PMC7210009

[B49] Park K. C. Geleta B. Leck L. Y. W. Paluncic J. Chiang S. Jansson P. J. (2020b). Thiosemicarbazones suppress expression of the c-Met oncogene by mechanisms involving lysosomal degradation and intracellular shedding. J. Biol. Chem. 295 (2), 481–503. 10.1074/jbc.RA119.011341 31744884 PMC6956523

[B50] Park K. C. Menezes S. V. Kalinowski D. S. Sahni S. Jansson P. J. Kovacevic Z. (2018). Identification of differential phosphorylation and sub-cellular localization of the metastasis suppressor, NDRG1. Biochim. Biophys. Acta Mol. Basis Dis. 1864 (8), 2644–2663. 10.1016/j.bbadis.2018.04.011 29679718

[B51] Park K. C. Paluncic J. Kovacevic Z. Richardson D. R. (2020c). Pharmacological targeting and the diverse functions of the metastasis suppressor, NDRG1, in cancer. Free Radic. Biol. Med. 157, 154–175. 10.1016/j.freeradbiomed.2019.05.020 31132412

[B52] Paul B. T. Manz D. H. Torti F. M. Torti S. V. (2017). Mitochondria and Iron: current questions. Expert Rev. Hematol. 10 (1), 65–79. 10.1080/17474086.2016.1268047 27911100 PMC5538026

[B53] Puig S. Ramos-Alonso L. Romero A. M. Martinez-Pastor M. T. (2017). The elemental role of iron in DNA synthesis and repair. Metallomics 9 (11), 1483–1500. 10.1039/c7mt00116a 28879348

[B54] Ren G. F. Tang L. Yang A. Q. Jiang W. W. Huang Y. M. (2014). Prognostic impact of NDRG2 and NDRG3 in prostate cancer patients undergoing radical prostatectomy. Histol. Histopathol. 29 (4), 535–542. 10.14670/HH-29.10.535 24222185

[B55] Renassia C. Peyssonnaux C. (2019). New insights into the links between hypoxia and iron homeostasis. Curr. Opin. Hematol. 26 (3), 125–130. 10.1097/MOH.0000000000000494 30855332 PMC6467554

[B56] Rohlenova K. Sachaphibulkij K. Stursa J. Bezawork-Geleta A. Blecha J. Endaya B. (2017). Selective disruption of respiratory supercomplexes as a new strategy to suppress Her2high breast cancer. Antioxid. Redox Signal 26 (2), 84–103. 10.1089/ars.2016.6677 27392540 PMC5206771

[B57] Sahni S. Park K. C. Kovacevic Z. Richardson D. R. (2019). Two mechanisms involving the autophagic and proteasomal pathways process the metastasis suppressor protein, N-myc downstream regulated gene 1. Biochim. Biophys. Acta Mol. Basis Dis. 1865 (6), 1361–1378. 10.1016/j.bbadis.2019.02.008 30763642

[B58] Sandoval-Acuna C. Fuentes-Retamal S. Guzman-Rivera D. Peredo-Silva L. Madrid-Rojas M. Rebolledo S. (2016). Destabilization of mitochondrial functions as a target against breast cancer progression: role of TPP(+)-linked-polyhydroxybenzoates. Toxicol. Appl. Pharmacol. 309, 2–14. 10.1016/j.taap.2016.08.018 27554043

[B59] Sandoval-Acuna C. Torrealba N. Tomkova V. Jadhav S. B. Blazkova K. Merta L. (2021). Targeting mitochondrial iron metabolism suppresses tumor growth and metastasis by inducing mitochondrial dysfunction and mitophagy. Cancer Res. 81 (9), 2289–2303. 10.1158/0008-5472.CAN-20-1628 33685989

[B60] Sang Y. Kong P. Zhang S. Zhang L. Cao Y. Duan X. (2020). SGK1 in human cancer: emerging roles and mechanisms. Front. Oncol. 10, 608722. 10.3389/fonc.2020.608722 33542904 PMC7851074

[B61] Sevinsky C. J. Khan F. Kokabee L. Darehshouri A. Maddipati K. R. Conklin D. S. (2018). NDRG1 regulates neutral lipid metabolism in breast cancer cells. Breast Cancer Res. 20 (1), 55. 10.1186/s13058-018-0980-4 29898756 PMC6001025

[B62] Sheftel A. D. Mason A. B. Ponka P. (2012). The long history of iron in the Universe and in health and disease. Biochim. Biophys. Acta 1820 (3), 161–187. 10.1016/j.bbagen.2011.08.002 21856378 PMC3258305

[B63] Shehadeh-Tout F. Milioli H. H. Roslan S. Jansson P. J. Dharmasivam M. Graham D. (2023). Innovative thiosemicarbazones that induce multi-modal mechanisms to down-regulate estrogen-progesterone-androgen- and prolactin-receptors in breast cancer. Pharmacol. Res. 193, 106806. 10.1016/j.phrs.2023.106806 37244387

[B64] Shen L. Qu X. Li H. Xu C. Wei M. Wang Q. (2018). NDRG2 facilitates colorectal cancer differentiation through the regulation of Skp2-p21/p27 axis. Oncogene 37 (13), 1759–1774. 10.1038/s41388-017-0118-7 29343851 PMC5874257

[B65] Shi X. H. Larkin J. C. Chen B. Sadovsky Y. (2013). The expression and localization of N-myc downstream-regulated gene 1 in human trophoblasts. PLoS One 8 (9), e75473. 10.1371/journal.pone.0075473 24066183 PMC3774633

[B66] Stevens R. G. Jones D. Y. Micozzi M. S. Taylor P. R. (1988). Body iron stores and the risk of cancer. N. Engl. J. Med. 319 (16), 1047–1052. 10.1056/NEJM198810203191603 3173433

[B67] Torti S. V. Torti F. M. (2013). Iron and cancer: more ore to be mined. Nat. Rev. Cancer 13 (5), 342–355. 10.1038/nrc3495 23594855 PMC4036554

[B68] Truksa J. Dong L. F. Rohlena J. Stursa J. Vondrusova M. Goodwin J. (2015). Mitochondrially targeted vitamin E succinate modulates expression of mitochondrial DNA transcripts and mitochondrial biogenesis. Antioxid. Redox Signal 22 (11), 883–900. 10.1089/ars.2013.5594 25578105

[B69] Villodre E. S. Gong Y. Hu X. Huo L. Yoon E. C. Ueno N. T. (2020). NDRG1 expression is an independent prognostic factor in inflammatory breast cancer. Cancers (Basel) 12 (12), 3711. 10.3390/cancers12123711 33321961 PMC7763268

[B70] Villodre E. S. Hu X. Eckhardt B. L. Larson R. Huo L. Yoon E. C. (2022). NDRG1 in aggressive breast cancer progression and brain metastasis. J. Natl. Cancer Inst. 114 (4), 579–591. 10.1093/jnci/djab222 34893874 PMC9002276

[B71] Wang J. Yin D. Xie C. Zheng T. Liang Y. Hong X. (2014). The iron chelator Dp44mT inhibits hepatocellular carcinoma metastasis via N-Myc downstream-regulated gene 2 (NDRG2)/gp130/STAT3 pathway. Oncotarget 5 (18), 8478–8491. 10.18632/oncotarget.2328 25261367 PMC4226698

[B72] Yu C. Hao X. Zhang S. Hu W. Li J. Sun J. (2019). Characterization of the prognostic values of the NDRG family in gastric cancer. Ther. Adv. Gastroenterol. 12, 1756284819858507. 10.1177/1756284819858507 PMC6647212 31384305

[B73] Yu Y. Kovacevic Z. Richardson D. R. (2007). Tuning cell cycle regulation with an iron key. Cell. Cycle 6 (16), 1982–1994. 10.4161/cc.6.16.4603 17721086

[B74] Zhai Z. Mu T. Zhao L. Li Y. Zhu D. Pan Y. (2022). MiR-181a-5p facilitates proliferation, invasion, and glycolysis of breast cancer through NDRG2-mediated activation of PTEN/AKT pathway. Bioengineered 13 (1), 83–95. 10.1080/21655979.2021.2006974 34951340 PMC8805873

[B75] Zhou J. Jiang Y. Zhao J. Zhang H. Fu J. Luo P. (2020). Dp44mT, an iron chelator, suppresses growth and induces apoptosis via RORA-mediated NDRG2-IL6/JAK2/STAT3 signaling in glioma. Cell. Oncol. (Dordr) 43 (3), 461–475. 10.1007/s13402-020-00502-y 32207044 PMC12990703

[B76] Zhou L. Zhao B. Zhang L. Wang S. Dong D. Lv H. (2018). Alterations in cellular iron metabolism provide more therapeutic opportunities for cancer. Int. J. Mol. Sci. 19 (5), 1545. 10.3390/ijms19051545 29789480 PMC5983609

