# Supplementary figures and images for: NDRG1 acts as an oncogene in triple-negative breast cancer and its loss sensitizes cells to mitochondrial iron chelation
Source: Front Pharmacol. 2024 Jun 25;15:1422369. doi: 10.3389/fphar.2024.1422369 (PMC11231402; doi:10.3389/fphar.2024.1422369)

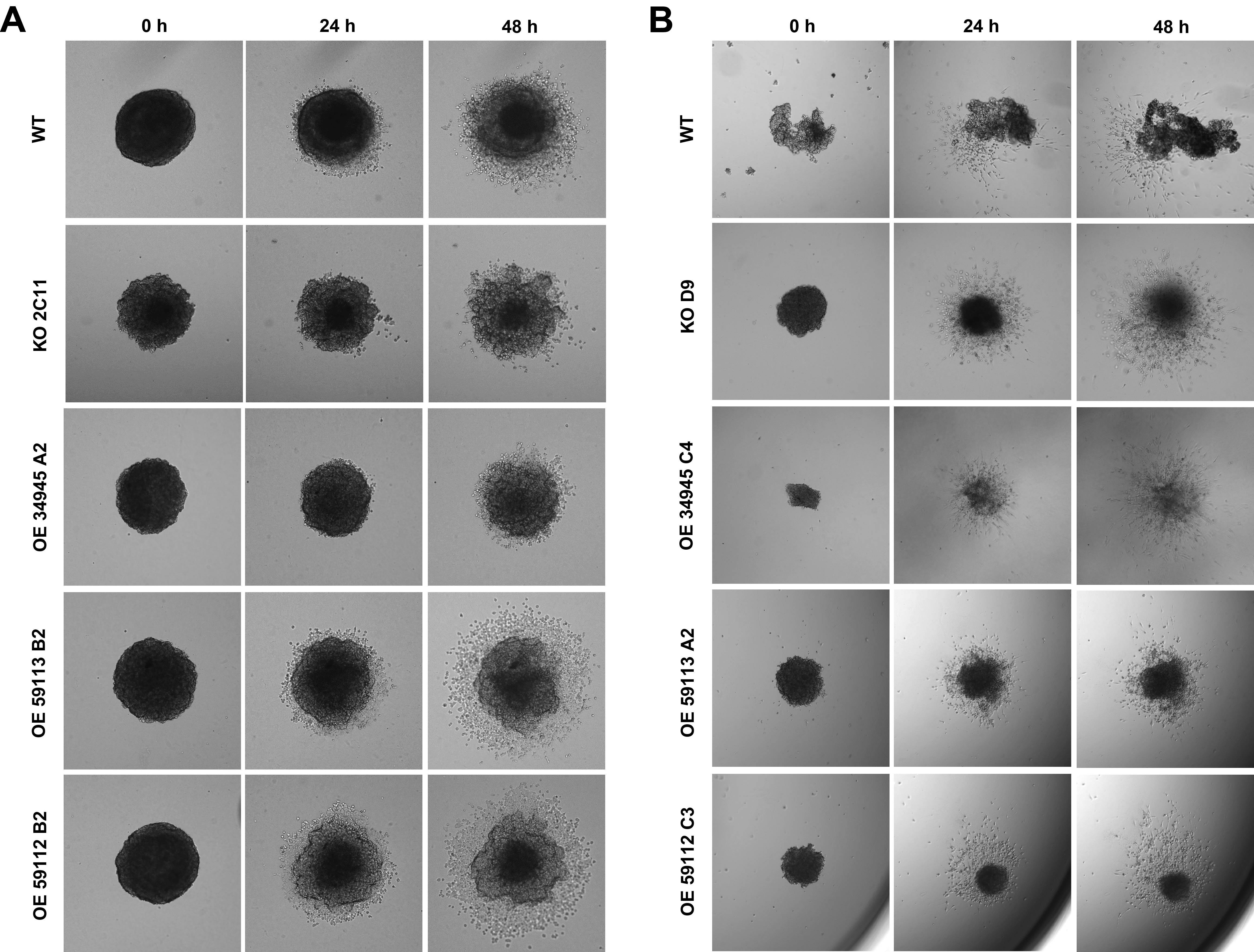

Supplement: Supplementary file 1 [file Image3.JPEG]

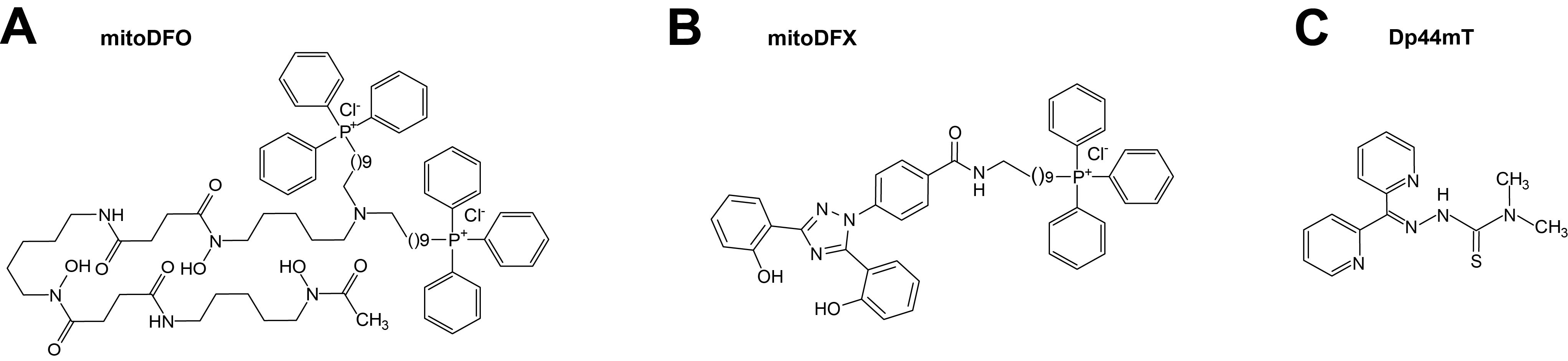

Supplement: Supplementary file 3 [file Image1.JPEG]

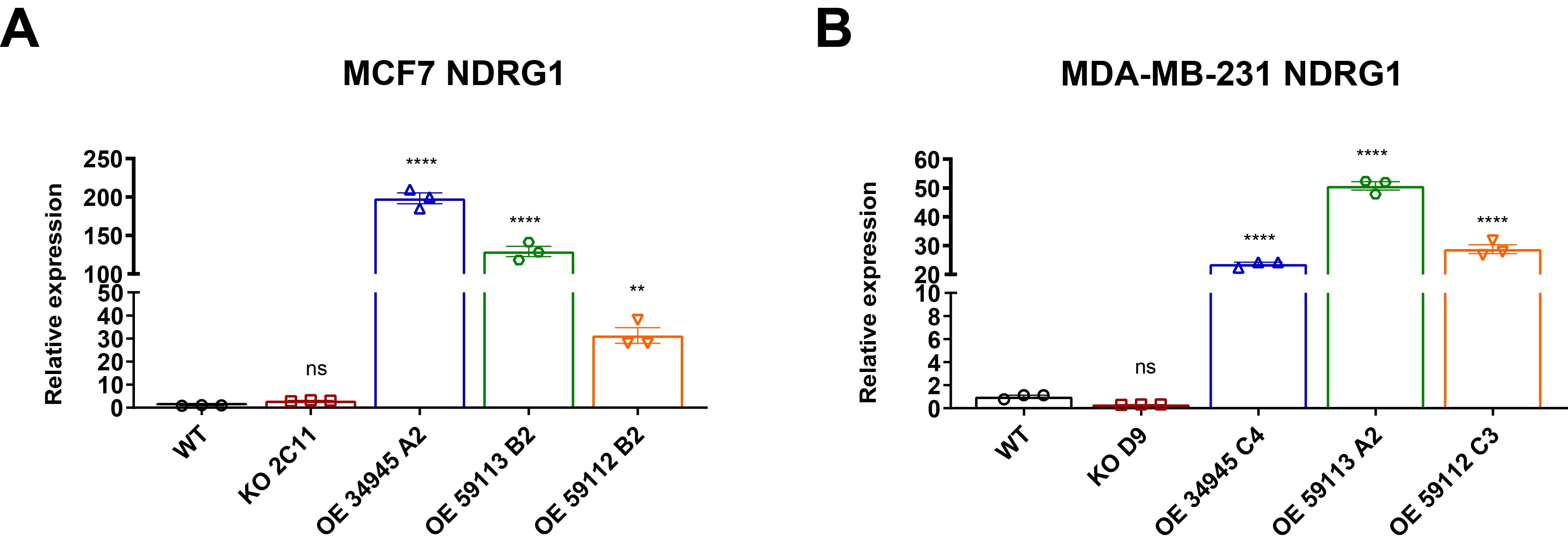

Supplement: Supplementary file 4 [file Image2.JPEG]
