# Supplementary material for: NDRG1 acts as an oncogene in triple-negative breast cancer and its loss sensitizes cells to mitochondrial iron chelation
Source: Front Pharmacol. 2024 Jun 25;15:1422369. doi: 10.3389/fphar.2024.1422369 (PMC11231402; doi:10.3389/fphar.2024.1422369)
Supplement: Supplementary file 2 [file Table1.docx]

**Supplementary Tables**

*1. Western Blot Antibodies*

| **Antibody** | **Manufacturer** | **Catalogue #** |
| --- | --- | --- |
| NDRG1 | Thermo Scientific | 42-6200 |
| NDRG2 | Santa Cruz Biotech | sc-376202 |
| NDRG3 | Santa Cruz Biotech | sc-514561 |
| NDRG4 | Santa Cruz Biotech | sc-514144 |
| GSK-3α | Cell Signaling Technology | 4337 |
| GSK-3β | Cell Signaling Technology | 12456 |
| p-NDRG1 Ser330 | Cell Signaling Technology | 3506 |
| p-NDRG1 Thr346 | Cell Signaling Technology | 5482 |
| p-GSK 3α (Ser21) | Cell Signaling Technology | 9316 |
| p- GSK 3β (Ser9) | Cell Signaling Technology | 5558 |
| p-GSK 3α/ β (Ser21/9) | Cell Signaling Technology | 8566 |
| Actin-HRP | Santa Cruz Biotechnology | Sc-47778 HRP |

*2. qPCR primers*

| **Primer** | **Manufacturer** | **Sequence** |
| --- | --- | --- |
| NDRG1 | Generi Biotech | F 5’-CCAACAAAGACCACTCTCCTC-3’  R 5’-CCATGCCCTGCACGAAGTA-3’ |
| NDRG2 | Generi Biotech | F 5’ - ATCTTGCTTCCAGCCACTGTT-3’  R 5’- GTCCGGGTGGTTAAGAGCATA-3’ |
| NDRG3 | Generi Biotech | F 5’- CGCGCCTCAGAGTTACTGAT-3’  R 5’- AGTGCTGGGTGATCTCTTGC-3’ |
| NDRG4 | Generi Biotech | F 5’- TCTCCGGCCTAACTAGCACT-3’  R 5’- GGGTGCATTATCCCCAACCA-3’ |
| RPLP0 | Invitrogen | F 5’-ATCACAGAGGAAACTCTGCATTCTCG-3’  R 5’-GATAGAATGGGGTACTGATGCAACAGTT-3’ |

*3. Confocal Microscopy Antibodies*

| **Antibody** | **Manufacturer** | **Catalogue #** |
| --- | --- | --- |
| NDRG1 | Thermo Fisher Scientific | 42-6200 |
| goat anti-rabbit AF488 | Life Technologies | A11034 |

*4. Cloning oligos for KO generation*

| **Name** | **Manufacturer** | **Sequence** |
| --- | --- | --- |
| NDRG1 CPF1 ex 4 F | Generi Biotech | 5’-AGATCGCCACTCTCTTGGGAACCGGCTAATTTCTACTCTTGTAGATCCTTGGGAGTCCCACACAGCGTGAATTTCTACTCTTGTAGATAGGAAGCTCTCTTCTCATCTGGC-3’ |
| NDRG1 CPF1 ex 4 R | Generi Biotech | 5’-AAAAGCCAGATGAGAAGAGAGCTTCCTATCTACAAGAGTAGAAATTCACGCTGTGTGGGACTCCCAAGGATCTACAAGAGTAGAAATTAGCCGGTTCCCAAGAGAGTGGCG-3’ |
| NDRG1 seq ex 4 F | Generi Biotech | 5’-AACAGCCCCAGGAAGTCCCAGG-3’ |
| NDRG1 seq ex 4 R | Generi Biotech | 5’-CCTAGCCCTGGTCCTTGGAGTGG-3’ |

*5. Cloning oligos for NDRG1 OE*

| **Name** | **Manufacturer** | **Sequence** |
| --- | --- | --- |
| NDRG159112cFBamHI | Thermo Scientific | 5’-CAGTTACATTGGATCCACCATGCAGGAGATCACCCAGC-3’ |
| NDRG159113cFBamHI | Thermo Scientific | 5’-CAGTTACATTGGATCCACCATGAACCACAAAACCTGCTACAA-3’ |
| NDRG34945cFBamHI | Thermo Scientific | 5’-CAGTTACATTGGATCCACCATGTCTCGGGAGATGCAGGATGT-3’ |
| NDRG1allcRNotI | Thermo Scientific | 5’-CTCGAATTCGCGGCCGCCTAGCAGGAGACCTCCATGGACT-3’ |
